# A Multi-Center Clinical Study to Harvest and Characterize Circulating Tumor Cells from Patients with Metastatic Breast Cancer Using the Parsortix^®^ PC1 System

**DOI:** 10.3390/cancers14215238

**Published:** 2022-10-26

**Authors:** Evan N. Cohen, Gitanjali Jayachandran, Richard G. Moore, Massimo Cristofanilli, Julie E. Lang, Joseph D. Khoury, Michael F. Press, Kyu Kwang Kim, Negar Khazan, Qiang Zhang, Youbin Zhang, Pushpinder Kaur, Roberta Guzman, Michael C. Miller, James M. Reuben, Naoto T. Ueno

**Affiliations:** 1 Department of Hematopathology Research, Division of Pathology and Laboratory Medicine, The University of Texas MD Anderson Cancer Center, Houston, TX 77030, USA; 2 Division of Gynecologic Oncology, Department of Obstetrics and Gynecology, Wilmot Cancer Institute, University of Rochester Medical Center, Rochester, NY 14620, USA; 3 Department of Medicine-Hematology and Oncology, Robert H Lurie Comprehensive Cancer Center, Feinberg School of Medicine, Northwestern University, Chicago, IL 60611, USA; 4 USC Breast Cancer Program, Keck School of Medicine, Norris Comprehensive Cancer Center, University of Southern California, Los Angeles, CA 90033, USA; 5 Department of Pathology, Breast Cancer Analysis Laboratory, Keck School of Medicine, Norris Comprehensive Cancer Center, University of Southern California, Los Angeles, CA 90033, USA; 6 ANGLE Clinical Studies, ANGLE Europe Limited, Guildford, Surrey GU2 7AF, UK; 7 Department of Breast Medical Oncology, The University of Texas MD Anderson Cancer Center, Houston, TX 77030, USA

**Keywords:** circulating tumor cells, neoplastic cells, circulating, neoplasms/diagnosis, circulating/pathology, biopsy, breast neoplasms/pathology, biomarkers, tumor, blood, liquid biopsy

## Abstract

**Simple Summary:**

There is a great need to understand the cellular and molecular characteristics of cancer when access to the tumor is limited. Circulating tumor cells (CTCs) captured from the blood of cancer patients may serve as a surrogate source of tumor material. However, the only FDA-cleared CTC assay has been limited to counting CTC in blood and and lack further characterization of the CTCs. In this study, we tested the Parsortix^®^ PC1 System that captures and harvests a wide range of CTCs from peripheral blood that are amenable for further evaluation. The device was assessed in a large, multicenter clinical trial including patients with metastatic breast cancer and healthy volunteers, with enriched CTC evaluated by 4 downstream techniques commonly available in clinical laboratories. The data generated from this study was used to support FDA clearance for the Parsortix System.

**Abstract:**

Circulating tumor cells (CTCs) captured from the blood of cancer patients may serve as a surrogate source of tumor material that can be obtained via a venipuncture (also known as a liquid biopsy) and used to better understand tumor characteristics. However, the only FDA-cleared CTC assay has been limited to the enumeration of surface marker–defined cells and not further characterization of the CTCs. In this study, we tested the ability of a semi-automated device capable of capturing and harvesting CTCs from peripheral blood based on cell size and deformability, agnostic of cell-surface markers (the Parsortix^®^ PC1 System), to yield CTCs for evaluation by downstream techniques commonly available in clinical laboratories. The data generated from this study were used to support a De Novo request (DEN200062) for the classification of this device, which the FDA recently granted. As part of a multicenter clinical trial, peripheral blood samples from 216 patients with metastatic breast cancer (MBC) and 205 healthy volunteers were subjected to CTC enrichment. A board-certified pathologist enumerated the CTCs from each participant by cytologic evaluation of Wright-Giemsa-stained slides. As proof of principle, cells harvested from a concurrent parallel sample provided by each participant were evaluated using one of three additional evaluation techniques: molecular profiling by qRT-PCR, RNA sequencing, or cytogenetic analysis of HER2 amplification by FISH. The study demonstrated that the Parsortix^®^ PC1 System can effectively capture and harvest CTCs from the peripheral blood of MBC patients and that the harvested cells can be evaluated using orthogonal methodologies such as gene expression and/or Fluorescence In Situ Hybridization (FISH).

## 1. Introduction

Circulating tumor cells (CTCs) are carcinoma cells which migrate through the intracellular matrix, actively enter the circulation through endothelial cells, presumably of capillaries and venules, and are disseminated through the bloodstream. Some CTCs survive to attach and penetrate the endothelial cells of capillaries and venules in distant organs, thereby forming metastases in these distant organs. Hence, CTCs are characteristically found in the blood of patients with metastases. The potential of a liquid biopsy to procure tumor cells before and during treatment in a non-invasive fashion has generated substantial interest in its use in oncology research and clinical practice. However, isolating CTCs from blood is inherently challenging, which has limited the use of CTCs in the clinical setting [1].

CTCs are usually rare, representing a minuscule fraction of the cells present in a blood sample. Consequently, the number of CTCs isolated from a single-tube blood draw (5–10 mL of peripheral blood) is typically very low, frequently being from 1 to 15 cells. Nonetheless, these cells provide valuable data: several lines of evidence have confirmed that the detection of CTCs represents an innovative and reliable tool to predict disease progression and overall survival in patients with metastatic breast cancer (MBC) [2,3,4,5,6,7]. Furthermore, the enumeration of CTCs at different time points during treatment is considered a reliable surrogate marker of treatment response and a potential alternative form of non-invasive monitoring of response to therapy [5,6,7].

Many technologies have been developed to isolate, enumerate, and characterize CTCs [1,8,9,10]. Of these, the CELLSEARCH^®^ System (Menarini-Silicon Biosystems, Huntingdon Valley, PA, USA) is the only CTC device cleared by the U.S. Food and Drug Administration (FDA). The CELLSEARCH^®^ System was cleared specifically for the enumeration of CTCs from the blood of patients with metastatic breast, colorectal, and prostate cancer [11].

The CELLSEARCH^®^ System captures CTCs based on immune affinity using antibodies specific to epithelial cell adhesion molecule (EpCAM). This cell-surface protein is expressed by many CTC subsets but is neither specific to CTCs nor is it universally expressed by all CTCs. Antibodies against surface EpCAM are routinely used to capture CTCs from blood, but such an approach is inherently limited to tumor cells with epithelial differentiation.

Cancer development frequently involves a transition of cells from an epithelial phenotype to a mesenchymal phenotype (a process referred to as epithelial-to-mesenchymal transition, or EMT), which results in the downregulation of EpCAM expression [12,13] and is associated with tumor-initiating potential [14,15]. During this switch to EMT, epithelial cells undergo upregulation of mesenchymal gene expression patterns and downregulation of epithelial genes. Furthermore, epithelial cells lose the ability to form streamlined cell–cell connections and cell polarity due to the restructuring of their cytoskeleton. Consequently, individual cells gain increased motility potential and an invasive phenotype [16]. EpCAM-based methods, therefore, fail to efficiently capture mesenchymal cells, leading to the selective isolation of CTC phenotypes that may not be representative of most cells being shed from a tumor that have the ability to establish themselves and grow at a distal site. In addition, not all epithelial cancer cells express EpCAM [17].

Antibody-based capture methods may also impact further characterization, such as gene expression analyses [18]. As gene expression, by nature, reflects external signals received by cells and consequent signaling pathways within them, the interaction of capture antibodies with the cell surface may alter gene expression data obtained from CTCs captured using immune-affinity enrichment methods. Altogether, these limitations underscore an unmet need for the agnostic enrichment of intact CTCs that can be used in clinically meaningful downstream analyses.

The Parsortix^®^ PC1 System is a semi-automated device based on based microfluidic technology that enables the capture and harvest of rare cells (e.g., CTCs) from peripheral blood based on cell size and deformability [19,20,21,22,23,24,25]. It addresses several issues encountered with current CTC capture technologies because it does not use antibodies or other cell-surface affinity agents to capture the target cells. The isolation/capture mechanism employed by the system is a purely physical method rather than a chemical or biological one, making it epitope independent and consequently agnostic to cellular phenotypes [21,22,23] and able to capture cells with mesenchymal features.

This multi-center clinical study, entitled “Harvest of Circulating Tumor Cells (CTCs) from Patients with Metastatic Breast Cancer (MBC) Using the Parsortix^®^ PC1 System” (the ANG-002 HOMING study; ClinicalTrials.gov identifier: NCT03427450), was designed and conducted to demonstrate that the Parsortix^®^ PC1 System can capture and harvest CTCs from the peripheral blood of patients with MBC and that the CTCs harvested by the system can be used for subsequent downstream evaluation. Cytology evaluation, quantitative polymerase chain reaction (PCR), fluorescence in situ hybridization (FISH), and RNA-seq were chosen as representative downstream evaluation methods, covering a range of molecular, histopathological, and cytomorphological techniques currently used in clinical laboratories. The results from the HOMING study demonstrated that CTCs can indeed be harvested from the peripheral blood of patients with MBC and utilized in subsequent downstream analysis methods. The data generated under this study were included in a De Novo request for classification of the Parsortix^®^ PC1 System (DEN200062) as a Class II prescription device, and the FDA granted the request on 24 May 2022 (https://www.accessdata.fda.gov/cdrh_docs/pdf20/DEN200062.pdf, accessed on 20 October 2022).

## 2. Materials and Methods

### 2.1. Ethical Conduct of the Study

The ANG-002 HOMING study was an IRB-approved prospective clinical trial registered with ClinicalTrials.gov (identifier: NCT03427450) and sponsored by ANGLE Europe Limited (Guildford, UK), the manufacturer of the Parsortix^®^ PC1 System. The study involved the collection of whole-blood samples from patients with MBC (either women with newly diagnosed MBC who were about to start a new line of therapy of any type to treat and/or manage their disease or those with currently progressive or recurrent MBC) as well as from a control population of healthy female volunteers (HVs) consisting of women who self-declared no prior/current history of cancer and no known history of breast disease. All study participants provided informed consent before participation in the study. All laboratory testing was performed by operators blinded to the clinical status of the participants.

Participants were enrolled, and samples were collected and processed at four institutions: The University of Texas MD Anderson Cancer Center, Houston, TX, USA; University of Southern California, Los Angeles, CA, USA; University of Rochester Medical Center, Rochester, NY, USA; and Northwestern University, Chicago, IL, USA. The study was conducted with the approval of each institution’s institutional review board and in accordance with the Declaration of Helsinki. A study-specific online database was designed, constructed, and maintained using the University of Rochester Medical Center’s REDCap system [26,27].

### 2.2. Blood Collection and Processing

Each participant provided between ~7 mL and 23 mL of whole blood collected specifically for this study into one 3 mL K_2_EDTA tube followed by two 10 mL K_2_EDTA tubes at a single time point. For patients with MBC, blood was collected before the initiation of their new therapy and a minimum of 7 days after the last administration of any previous cytotoxic treatment, either from venipuncture or through an existing port. For patients continuing an existing oral hormonal and/or targeted immunotherapy in addition to starting a new treatment, blood was drawn before the next administration of their oral hormonal therapy and/or immunotherapy and before the initiation of their new therapy treatment. For HVs, blood was collected via venipuncture on the day of study.

For the capture of CTCs, all samples were processed using the Parsortix^®^ PC1 System within 8 h of collection. First, the blood volume in each of the K_2_EDTA tubes was estimated using an engineering ruler. The initial portion of blood collected into the 3 mL K_2_EDTA tube immediately following the venipuncture or the port flushing was used for a complete blood count with leukocyte differential testing. For the two 10 mL K_2_EDTA tubes, a minimum combined volume of ≥5 mL of blood was required for processing using the Parsortix^®^ PC1 System equipped with a Parsortix GEN3D6.5 Cell Separation Cassette. If both tubes contained a combined total of <5 mL of blood, the participant was considered non-evaluable, and the blood was discarded. If only one of the 10 mL K_2_EDTA tubes contained ≥5 mL of blood, then only the blood in that tube was processed for the primary cytological evaluation. If both tubes had <5 mL of blood, but the combined volume of blood in both tubes was ≥5 mL, then the blood from the two tubes was combined in a 10 mL K_2_EDTA tube into ≥5 mL of blood that was processed for the primary cytological evaluation. If both 10 mL K_2_EDTA tubes had ≥5 mL of blood, then the tube with the higher volume of blood was processed for the primary cytological evaluation, and the other tube was processed for one of the exploratory evaluations (qPCR, FISH, or RNA-seq).

Information about samples was blinded from the processors, and no follow-up information was collected for any participants. The population of cells captured from each blood sample by the Parsortix^®^ PC1 System was harvested directly from the cell separation cassettes (each harvest consisting of a total volume of 210 µL of phosphate-buffered saline) into collection vessels and used for downstream processing and characterization. 

### 2.3. Downstream Characterization

#### 2.3.1. Primary Evaluation

For all participants, the cells harvested from the 10 mL K_2_EDTA tube containing the larger volume of blood were subjected to cytomorphological evaluation by a qualified pathologist (JDK) to determine the presence and number of observable CTCs.

##### Cytology Processing

Following enrichment, cells were harvested into a 1.5 mL microfuge tube containing 60 µL of fetal bovine serum (FBS). The harvested cells suspended in FBS were pipetted into a Cytospin 4 Cytofunnel assembly (ThermoFisher Scientific, Waltham, MA, USA) containing a positively charged glass Cytoslide (ThermoFisher Scientific). The slide assembly was cytocentrifuged at 800 rpm for 3 min on low acceleration, and the slide was removed from the assembly and allowed to air-dry at room temperature for 1 min. The air-dried slide was then submersed in 100% methanol for 1 min, removed, gently tapped at the edge on a paper towel to remove any excess methanol, and allowed to air-dry at room temperature for 30 min. The fixed slides were stored at room temperature until shipment weekly to the designated central testing laboratory located at the MD Anderson Cancer Center.

At the laboratory, the slides underwent Wright-Giemsa staining on an automated stainer, and examination by a qualified pathologist (JDK) with expertise in blood evaluation and cytopathology who identified and enumerated CTCs using conventional cytomorphological criteria of malignancy, which included: increased nuclear-to-cytoplasmic ratio, cellular pleomorphism, large size relative to white blood cells, irregular nuclear membrane, chromatin structure, nuclear hyperchromasia, cytoplasmic vacuoles, cellular aggregates (≥2 cells). The stained slides were evaluated by light microscopy, and the cells that had been cytomorphologically identified as CTCs by the qualified pathologist were photographed, identified, and counted. Cells without definite features of malignancy but distinct from usual peripheral blood–formed elements (e.g., neutrophils, immature granulocytic precursors, monocytes, nucleated red blood cells) were not counted as CTCs. Naked nuclei or cell fragments were not evaluated. Samples in which technical artifacts caused substantial deformation of peripheral blood elements to an extent that compromised their morphological evaluation were considered unsatisfactory.

#### 2.3.2. Exploratory Evaluations

For participants for whom both 10 mL K_2_EDTA tubes contained ≥5 mL of blood, the cells harvested from the second tube were subjected to one of the following exploratory evaluations.

##### Gene Expression Evaluation by qRT-PCR Processing

Cells captured in the cassette were harvested directly into a 2.0 mL microfuge tube and centrifuged at ~400× *g* for 5 min at 4 °C, and as much of the supernatant as possible was removed without disturbing the cell pellet. The cell pellet was resuspended in 320 µL of Qiagen buffer RLT containing 1% 2-mercaptoethanol. Lysates were stored at −80 °C until batch shipment to the designated central qPCR testing laboratory at The MD Anderson Cancer Center for gene expression analysis using quantitative reverse-transcriptase real-time PCR (qRT-PCR). Each lysate was evaluated for expression of the following genes using hydrolysis (TaqMan) probes (Bio-Rad Laboratories, Hercules, CA, USA): GAPDH and B2M (housekeeping genes), GYPA (a nucleated red blood cell marker), PTPRC (a white blood cell marker), EpCAM and KRT19 (epithelial cell markers), ERBB2 (a breast tumor marker), and TWIST1 and SNAI2 (mesenchymal cell markers). Each gene was analyzed in triplicate for every sample (including samples from patients, healthy volunteers, positive and negative controls), and 40 cycles of PCR were performed. PCR thermocycling and data acquisition were performed using the appropriate instrumentation and software, which automatically set the cycle threshold (Ct). The average of the three replicate Ct values for each gene target for each sample and for the positive and negative controls were used for the evaluations presented in this report. For all instances where a gene was undetectable after 40 cycles of PCR, a Ct value of 40.0 was assigned for analysis purposes. Aliquots of nuclease-free water were used as negative controls for the assay. The SUM149 triple-negative breast cancer cell line was selected as a positive control since it exhibits a partial EMT phenotype. Aliquots of SUM149 cell lysate were used as positive controls. They were expected to have positive expression for GAPDH, B2M, KRT19, EpCAM, ERBB2, TWIST1, and SNAI2 while lacking expression of the white blood cell marker PTPRC and the nucleated red blood cell marker GYPA. Expression results are shown as 40-Ct values so that increased values reflect increased expression, and undetectable values are represented as 0. Normalization was not used so that gene expression can be interpreted as expression per tube of blood since the number of captured CTCs is variable.

##### RNA-seq Processing

An aliquot of 200 μL of whole blood from the 10 mL K_2_EDTA tube was transferred directly into a 1.5 mL microfuge tube containing 1 mL of RNAlater RNA Stabilization Solution before processing of the blood sample on the Parsortix^®^ PC1 System. The remaining blood was processed on the Parsortix^®^ PC1 System, and the cells captured in the cassette were harvested directly into a 0.2 mL PCR tube. The harvest was centrifuged at ~400× *g* for 5 min at room temperature, and as much of the supernatant as possible was removed without disturbing the cell pellet. The cell pellet was resuspended in 10 μL of Agilent SideStep lysis and stabilization buffer. Both lysates (i.e., the aliquot of whole blood and the harvest) were stored at −80 °C until shipment to the designated central RNA-seq testing laboratory at the University of Southern California. The RNA-seq laboratory isolated RNA from the whole blood lysates using Ambion RiboPure blood kits for RNA. The cDNA generated was amplified and purified using 50 ng of the purified RNA obtained from each of the whole blood lysates and 2 µL from each harvest lysates using NuGEN Trio RNA-Seq kits. cDNA libraries were then prepared, amplified, refined, and filtered for each sample using NuGEN Trio RNA-seq kits. The quantity and quality of each library preparation were assessed using DNA quantitation by a Qubit fluorometer. DNA fragment size distribution was determined using an Agilent 2100 Bioanalyzer. Only the cDNA libraries from the Parsortix^®^ harvest samples (and not the whole blood aliquots) were evaluated as a part of this report. The cDNA libraries generated from the harvest samples were sequenced using an Illumina HiSeq 2500 at the University of Southern California Genomics Core with 2 × 125 bp paired-end reads. Data analysis was conducted by an experienced bioinformatician (DC) to determine the expression patterns of breast cancer–related genes.

The RNA-seq method described above was validated in whole-transcriptome profiling studies in CTC from patients with non-metastatic breast cancer (stage II-III). These studies’ procedures and results were previously reported [28,29], and the results demonstrated that CTCs from patients with MBC could be used to generate cDNA libraries of sufficient quantity and quality that enable whole-genome sequencing.

Salmon 1.5.0 mapped the HV and MBC Parsortix^®^ PC1 harvest sequencing data against the reference transcriptome V37 from Gencode [30]. Quantification output from Salmon is reported in transcripts per kilobase million (TPM), computed by dividing read counts by the length of each gene in kilobases to obtain reads per kilobase (RPK). All the RPK values in a sample are summed, and the summation is divided by 1,000,000 to provide a “per million” scaling factor. The RPK values are divided by the per million scaling factors to obtain the TPM value. The sum of all TPM values is the same in each sample, making it easier to compare the proportion of reads mapped to a gene in each sample. 

In contrast, with reads per kilobase million (RPKM) and fragments per kilobase million (FPKM), the sum of the normalized reads in each sample may be different, making it harder to compare models directly. In a second analysis, a listing of all unmapped reads (i.e., reads that did not map to the transcriptome) from the Salmon analysis was tabulated for each sample. Magic-BLAST 1.5.0 was used to compare 100,000 randomly sampled from each of the unmapped reads files to the genome ver GRCh38 from Gencode, the transcriptome V37 from Gencode, and a file with all rDNA sequences downloaded from GenBank. The purpose was to determine whether the unmapped reads from Salmon analysis map better to the genome or the rDNA, instead of the transcriptome, which would support gDNA contamination. A result can contain more than 100,000 entries because a read can map to more than one genomic feature in the reference.

##### HER2 FISH Processing

Cells captured in the cassette were harvested directly into a 1.5 mL microfuge tube containing 60 µL of FBS. The harvested cells suspended in FBS were pipetted into a Cytospin 4 Cytofunnel assembly with a positively charged glass Cytoslide. The slide assembly was cytocentrifuged at 800 rpm for 3 min on low acceleration, and the slide was removed from the body and allowed to air-dry at room temperature for 1 min. The air-dried slide was then submersed in 100% methanol for 15 min, removed, gently tapped at the edge on a paper towel to remove any excess methanol, and allowed to air-dry at room temperature for 30 min. The fixed slides were stored at ≤−20 °C until shipment to the designated central HER2 FISH testing laboratory at the University of Southern California for processing, as described elsewhere [31,32,33], using commercially available HER2 FISH reagents (Abbott PathVysion HER2 DNA Probes [PN 30-171060/1800], DAPI II Counterstain [PN 30-804861/8100], NP-40 [PN 30-804820/8100], 20X SSC [PN 805850], and Vysis FISH Pretreatment Reagent Kits [PN 32-801270]) and evaluated by a board-certified pathologist (MFP). The pathologist determined the presence or absence of cells showing human epidermal growth factor receptor 2 (HER2/ERBB2) gene amplification. Criteria for evaluating CTCs for HER2 gene amplification status by FISH involved an assessment of the nuclear morphology to distinguish tumor cells from normal leukocytes in the slide preparations, followed by an evaluation of the HER2 gene copy number and chromosome 17 centromere (CEP17) copy number in the tumor cells. The criteria used in distinguishing tumor cells from white blood cells are similar to the requirements described for the cytological evaluation of the peripheral blood cells with the Wright-Giemsa stain. These criteria of malignancy included an increased nucleus size, nuclear pleomorphism, increased size relative to white blood cells, irregular nuclear membranes, increased nuclear DAPI staining, uneven distribution of atomic chromatin (heterochromatin and euchromatin), and aggregation of multiple, large cells. Because the CTCs contained intact tumor cell nuclei, not 4-micron histology tissue sections through tumor cell nuclei, the average chromosome 17 number was considered a reflection of overall DNA ploidy status as well as chromosome 17 aneusomy. A sample was considered HER2-amplified if the HER2/CEP17 ratio was greater than 2 and HER2 gene copies were present in groups as observed in human breast cancer cell lines known to have HER2 gene amplification with HER2/ERBB2 gene copies arranged as aggregates in homogeneous staining regions [34] CTCs with increased HER2 gene copy number greater than 4 but also paired with individual chromosome 17 centromeres, as observed in human breast cancer cell lines that lack HER2 gene amplification and lack HER2 mRNA/protein overexpression, were evaluated as HER2-not-amplified.

## 3. Results and Discussion

### 3.1. Patient Characteristics

**Figure 1:**
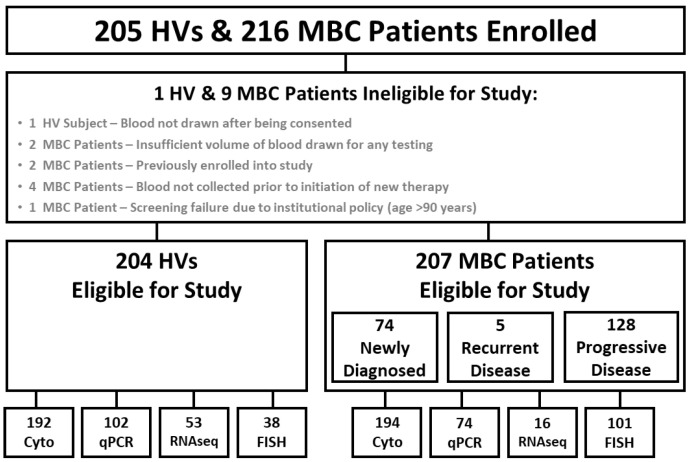
Study enrollment, eligibility, and evaluation. HV, healthy volunteer; MBC, metastatic breast cancer; Cyto, cytology evaluation; qPCR, quantitative polymerase chain reaction; FISH, fluorescence in situ hybridization.

A flow chart of participants enrolled in the ANG-002 HOMING study is shown in Figure 1. A total of 216 patients with metastatic breast cancer and 205 female healthy volunteers were enrolled at the four clinical study sites between April 2018 and February 2019. Nine (4.2%) of the patients with MBC and one (0.5%) of the HVs enrolled were ineligible for the study, leaving 207 eligible patients with MBC and 204 eligible HVs that were evaluable for one or more of the study endpoints. The HVs tended to be younger, healthier, and more racially diverse than the patients with MBC (Table 1).

As enrollment was open to any patient with MBC starting a new line of therapy, the patient population was split between those with newly diagnosed metastatic disease and others with progressive or recurrent disease at the time of the sample collection. Patient characteristics between these two cohorts were generally well-balanced. Overall, 27 (13.0%) of the patients with MBC had HER2-positive tumors (as determined from their medical records using the available HER2 IHC and/or FISH testing results on their primary and/or metastatic tumor tissue); only 4 (5.4%) of patients with newly diagnosed MBC had HER2-positive tumors, in contrast to 23 (17.3%, *p* = 0.014) of the patients with MBC with progressive and/or recurrent disease (Table 1). Despite these differences, the MBC patients enrolled in the ANG-002 study were representative of patients with MBC that would be seen in the general population [35].

### 3.2. Cytology Evaluation

To enumerate CTCs agnostic to protein expression bias, we used standard cytology techniques to identify cells based on longstanding morphologic features associated with malignancy. In validation studies, the cytocentrifugation (cytospin) method used to prepare slides for cytology and FISH evaluation showed significant cell loss for three cell lines (Figure S1). These results indicated that 37–51% of the cells harvested by the Parsortix^®^ PC1 System were lost due to the cytology slide preparation method and/or the Wright-Giemsa staining procedure (compared to harvesting the cells directly into 96-well plates).

**Figure 2:**
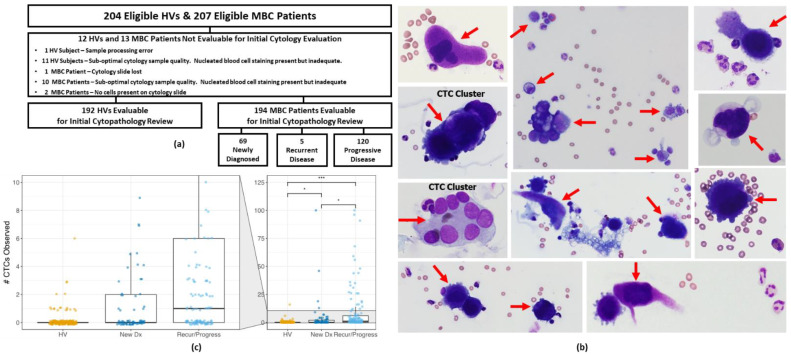
(**a**) Flow diagram for cytopathology evaluation in 207 eligible MBC patients. (**b**) Representative images of cells classified as CTCs (red arrows) from MBC patients that were harvested by the Parsortix PC1 system and deposited onto cytology slides by cytocentrifugation (images not to same scale) and Wright-Giemsa stained. (**c**) CTC numbers from the review of evaluable Wright-Giemsa-stained cytology slides. CTC, circulating tumor cell; HV, healthy volunteer; MBC, metastatic breast cancer. * *p* < 0.05, *** *p* < 0.001.

For the identification and enumeration of CTCs, the cells harvested from blood samples were fixed, Wright-Giemsa stained, and reviewed by a single pathologist; the resulting CTC prevalence rates in HVs and patients with MBC are shown in (Table 2). A flow diagram of the eligible subjects with evaluable cytology slides is shown in Figure 2a. In the 204 eligible HVs and 207 eligible patients with MBC, 12 (5.9%) and 13 (6.3%), respectively, did not produce evaluable slides for cytology examination, leaving a total of 192 HVs and 194 patients with MBC with evaluable Wright-Giemsa-stained cytology slides.

Examples of the Wright-Giemsa-stained CTCs are shown in Figure 2b, including harvested CTC clusters. Among the 194 patients with MBC with evaluable results, 94 (48.5%, 95% CI 41.5–55.4%) had one or more cells classified as CTCs, whereas 100 (51.5%, 95% CI 44.6–58.5%) had no cells classified as CTCs (Table 2 and Table 3, Figure 2c). Among the 192 HVs that had evaluable results, 173 (90.1%, 95% CI 85.1–93.6%) had no cells classified as CTCs, whereas 19 (9.9%, 95% CI 6.4–14.9%) had one or more cells classified as CTCs, representing a significantly lower rate of CTC detection compared to that of the patients with MBC (Fisher exact test *p* < 0.001, Table 3).

A significantly larger proportion of patients with MBC with recurring or progressive metastatic disease were found to have one or more CTCs compared to those with newly diagnosed metastatic disease (Table 3), which is consistent with what has been reported in the literature on CTCs in metastatic breast cancer [36]. Furthermore, CTC counts were significantly higher in patients with recurring/progressive disease (*p* = 0.019, Figure 2c).

Table 2 further summarizes the proportions of HVs and patients with MBC with CTCs observed on their cytology slides according to various demographic and clinical subgroups. Interestingly, among women with newly diagnosed MBC, approximately twice as many who were ≥ 57 years old (i.e., post-menopausal women) were observed to have CTCs compared to those who were < 57 years old (47.2% vs. 18.2%, respectively, *p* = 0.012).

There is also some evidence that the sample collection method had an impact on the CTC counts. A significantly larger proportion of the patients with MBC whose blood was drawn via a central port were observed to have CTCs compared with the patients whose blood was drawn via venipuncture (≥1 CTC: 94.3% vs. 38.4%, respectively, Fisher exact test *p* < 0.001, ≥5 CTCs: 62.9% vs. 13.8%, respectively, Fisher exact test *p* < 0.001, Table 2). This may be due to technical or procedural differences, volume, anatomic collection location, or patient population (a larger percentage of patients with progressive or recurrent disease had their blood samples collected via an installed port; Table 1). Previous reports have also shown that CTC levels can vary by anatomical location of cancer [37,38]. Additionally, peripheral blood drawn from antecubital veins has likely circulated through both lung and peripheral capillaries after egressing from tumors (from either primary, i.e., breast, or metastatic sites). In contrast, some blood from a central port comes directly from the tumor without first filtering through additional capillary beds. The Parsortix^®^ PC1 System enriches for cells that cannot pass through the ~6.5-μm critical gap of the separation cassette at 99 millibars of pressure (roughly equivalent to typical diastolic blood pressure); these same cells may likewise be unable to traverse the microcirculation of a capillary lumen. Another possible explanation for this observation is that patients with MBC with a central port indwelling usually receive intravenous treatments such as chemotherapy and thus may have a more aggressive disease compared to the wider population of patients with MBC. Progressive disease was noted in 31 (88.6%) of the 35 patients who had blood drawn from a central port and in only 94 (59.1%) of the 159 patients who had blood drawn via venipuncture (Fisher exact test *p* = 0.001, Table 2). Consequently, this subset of patients with MBC (i.e., those with a central port installed experiencing disease progression) is a specific population that would likely benefit from CTC evaluation.

In summary, cytologic evaluation showed that 48.5% (95% CI 41.5–55.4%) of all patients with MBC with evaluable staining results had one or more CTCs identified. Even though cytocentrifugation is a widely used method for depositing cells onto cytology slides and recent studies have shown that Parsortix-harvested cells deposited onto cytology slides via cytocentrifugation have effectively preserved cellular morphology [39], it is essential to note that this method caused significant loss (~57% on average) of the cells present in the Parsortix^®^ PC1 System harvests. Given this large observed cell loss, it is possible that a larger proportion of patients with MBC had CTCs present in their harvests, but these cells were simply not retained on the cytology slides. Alternative techniques to place cells on microscope slides without using the cytocentrifugation methods are under development. However, other downstream analysis techniques, such as gene expression analysis, may be able to utilize cells captured and harvested directly into a container without loss of the harvested cells potentially caused by subsequent manipulations of the harvested material.

### 3.3. Gene Expression by qRT-PCR

As described above, CTCs harvested directly for gene expression may serve as more effective biomarkers of CTCs. Although still subject to variation, gene expression analysis is relatively less subjective than image analysis and does not require deposition of the cells onto a slide. A subset of enrolled patients had a second blood tube processed by the Parsortix^®^ PC1 System, and the cells harvested were subjected to gene expression analysis by qRT-PCR (as described in this section) or RNA-seq (as described in the next section). The qRT-PCR assay used to evaluate gene markers for epithelial, mesenchymal and breast cancer cells was shown to be reliable and reproducible [40] and was able to detect a single epithelial cell that expressed *KRT19*, a single epithelial cell that expressed *ERRB2*, 10 cells that expressed *EPCAM*, or approximately 25–50 mesenchymal cells that expressed *SNAI2* and/or *TWIST1* (data not shown).

**Figure 3:**
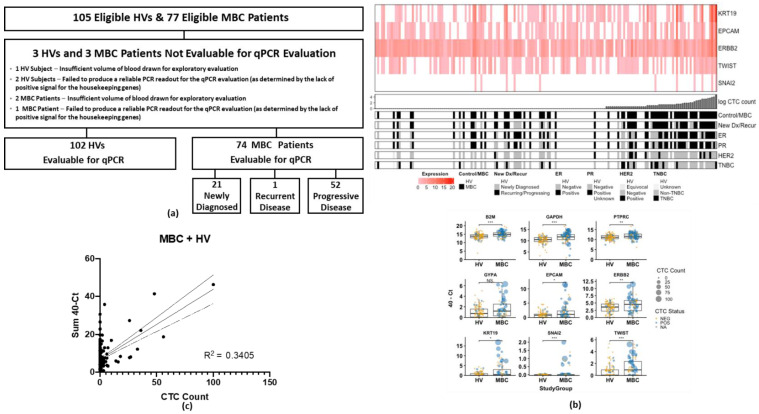
(**a**) Flow diagram for qPCR evaluation in eligible HV and MBC patients. (**b**) CTC count correlates with total CTC-related gene expression (40-Sum of KRT19, EPCAM, ERBB2, TWIST, and SNAI2 Ct values). Heat map and scatter plots of CTC gene expression show correlation of gene expression with the number of CTCs observed (**c**) Sum CTC related genes expression = 0.3701 ∗ CTC count + 6.771; R square = 0.3405, slope significantly different from zero (*p* < 0.0001). HV, healthy volunteer; MBC, metastatic breast cancer; qPCR, quantitative polymerase chain reaction. NS = *p* ≥0.05, * *p* < 0.05, ** *p* < 0.01, *** *p* < 0.001.

Figure 3a provides the flow chart for the 77 eligible patients with MBC and 105 eligible HVs where the second blood sample was used for qPCR regarding their eligibility for this exploratory evaluation. Only one of the lysates from the 75 patients with MBC and two lysates from the 104 HVs who had a sufficient volume of blood for processing failed to produce a reliable PCR readout (as determined by the lack of positive signal for the housekeeping genes), leaving a total of 74 patients with MBC and 102 HVs with evaluable qPCR results.

CTC gene expression from the qPCR evaluation and the corresponding enumeration from the cytology evaluation are summarized in Table 4. A total of 18 negative controls (nuclease-free water) and 18 positive controls (SUM149 cell lysate) were compared with the patient and HV samples. Using a Ct threshold of ≤35.0 for each of the genes to define positivity, none of the negative controls were positive for any of the genes, and 100% of the positive controls were positive for all of the genes (except the *GYPA* and *PTPRC* genes, as expected). In both the patients with MBC and HVs, 100% were positive for one or both of the housekeeping genes, 100% were positive for *PTPRC* (indicating the presence of white blood cells in all of the harvests), and <7% were positive for *GYPA* (indicating a low incidence of red blood cell contamination). As shown in Table 4, 52.7% of the patients with MBC were positive (Ct value ≤ 35.0) for at least one of the CTC-related genes (*KRT19*, *EPCAM*, *ERBB2*, *TWIST1*, and/or *SNAI2*), whereas only 19.6% of the HVs were positive for at least one of the CTC-related genes. Optimizing the Ct thresholds for each gene based on expression in the HVs increased the specificity of CTC genes at the expense of sensitivity (Table S1).

As shown in Figure 3b, control genes, including *GAPDH*, *B2M*, and *PTPRC* (CD45), were elevated in the samples from the MBC patients, suggesting both putative CTCs and white blood cells have a higher capture rate in samples from patients with MBC compared to samples from HVs. In the patients with MBC and HVs, respectively, 21.6% vs. 0% were positive for KRT19, 13.5% vs. 1.0% were positive for *EPCAM*, 47.3% vs. 19.6% were positive for ERBB2 (indicating a level of background expression for this gene), 5.4% vs. 1.0% were positive for TWIST1, and none were positive for SNAI2 (Table 4). Looking at the combined expression of only the KRT19, *EPCAM*, *TWIST1*, and/or *SNAI2* genes, only 1.0% of the HVs compared to 24.3% of the patients with MBC were positive for one or more of those cancer cell–related genes (Fisher exact test *p* < 0.001) (Table 4).

When compared to the number of CTCs identified using a cytomorphological review of the Wright-Giemsa stained cytology slides, the sum total expression of CTC-related genes (40-Ct of KRT19, *EPCAM*, *ERBB2*, *TWIST1*, and *SNAI2*) showed a general correlation with CTC enumeration, particularly at higher CTC burdens (R^2^ = 0.3405, Pearson’ rho = 0.569, *p* < 0.001, Spearman’s rho [less influenced by outliers] = 0.159, *p* = 0.034, Figure 3b,c). The discrepancy at lower CTC counts may reflect the utility of gene expression when morphology may be hard to distinguish or may reflect differences between parallel blood tubes (e.g., significant loss of harvested cells on the cytology slides due to cytocentrifugation slide preparation method). Furthermore, as seen in Table 4, only 1 (1.2%) of the 83 HVs with 0 CTCs identified on their cytology slides and none (0%) of the 16 HVs with ≥1 CTC identified on their cytology slides were positive for the combination of *KRT19*, *EPCAM*, *TWIST1*, and/or *SNAI2*, in contrast to 8 (25.8%) of the 31 patients with MBC with 0 CTCs identified on their cytology slides and 9 (22.5%) of the 40 patients with MBC with ≥1 CTC identified on their cytology slides (Table 4). This suggests that the orthogonal measures of morphology and gene expression could help increase detection specificity.

As seen in Figure 3b, samples enriched from patients with MBC had significantly elevated expression of *EPCAM* (Mann–Whitney U *p* = 0.016), *ERBB2* (*p* = 0.0025), *KRT19* (*p* = 0.049), and the mesenchymal cell–related genes SNAI2 (*p* < 0.001) and *TWIST1* (*p* < 0.001). Interestingly, when no CTCs were detected on the paired cytology slide, there was no difference in *EPCAM* or *KRT19* expression between patients with MBC and HVs, but *ERBB2*, *SNAI2*, and *TWIST1* remained elevated in the patient samples (Figure S2). Furthermore, samples from patients with MBC had elevated housekeeping control genes *B2M* and *GAPDH* independent of the detection of CTCs. In contrast, the white blood cell and red blood cell control genes *PTPRC* and *GYPA* were not elevated (Figure S2). Together, these results suggest that CTCs with mesenchymal features are less likely to be detected visually (or are more easily lost during the cytocentrifugation slide preparation method) and that adding the molecular characterization aided in their detection.

**Figure 4:**
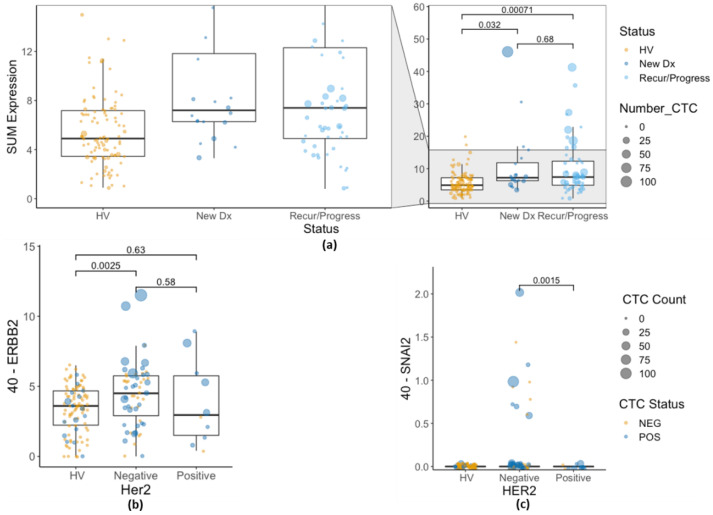
(**a**) Sum of CTC-related gene expression (40-Sum of EPCAM, KRT19, ERBB2, SNAI2, and TWIST Ct values) in HV, newly diagnosed MBC, and MBC with recurrence or progression. (**b**) ERBB2 expression in CTC-enriched samples by tissue HER2 status. (**c**) SNAI2 expression in CTC-enriched samples by tissue HER2 status. CTC, circulating tumor cell; HV, healthy volunteer; MBC, metastatic breast cancer.

Comparing samples from patients with newly diagnosed MBC and patients with recurrent or progressive MBC, there were no significant differences in total CTC gene expression (Figure 4a), in contrast to the stark differences in CTC counts between patients with MBC and HVs. However, compared to the HVs, *EPCAM*, *KRT19*, and *TWIST1* were significantly higher in patients with progressive/recurrent MBC (but not in newly diagnosed MBC); only *ERBB2* and *SNAI2* were elevated in both patient cohorts (Figure S3). This may be due to the relatively smaller sample size of patients in the newly diagnosed MBC patient cohort.

Patients with HER2-positive tumor tissue would be expected to have CTCs with elevated expression of *ERBB2* (the gene product for the HER2 protein). However, there was no significant difference in the *ERBB2* expression by enriched CTCs in patients with HER2-positive tumors compared to those with HER2-negative tumors, with the caveat that there were very few patients with HER2-positive MBC disease (40-Ct = 3.87 for patients with HER2-positive tumors vs. 40-Ct = 4.44 for patients with HER2-negative tumors, Wilcoxon signed rank test *p* = 0.4, Student’s *t*-test *p* = 0.5823, Figure 4b). Likewise, among the patients with MBC with detectable CTCs on their cytology slide, qPCR expression of *ERBB2* was nearly identical between HER2 groups (40-Ct = 4.44 for patients with HER2-positive tumors vs. 40-Ct = 4.53 for patients with HER2-negative tumors, Student’s *t*-test *p* = 0.936). However, in this study, all of the patients with MBC with HER2-positive tumors had recurring or progressing diseases at the time; none of the patients with HER2-positive tumors were newly diagnosed. Patients with MBC with HER2-negative tumors (who consequently did not receive HER2-targeted therapy) had higher expression of ERBB2 than HVs (*p* = 0.0025), whereas MBC patients with HER2-positive tumors did not (*p* = 0.63) (Figure 4b).

Furthermore, only patients with MBC with HER2-negative tumor tissue expressed SNAI2, although this difference was not statistically significant. Eleven patients with MBC with HER2-negative tumors showed expression of *SNAI2* by qPCR, while no patients with MBC with HER2-positive tumors and no HVs showed *SNAI2* expression (Fisher exact test *p* = 0.3455); average *SNAI2* expression was higher in the HER2-negative cohort (Figure 4c). Others have noted that HER2 expression can drive EMT [41,42,43,44,45,46], including EMT in CTCs [47], and can predict the presence of CTCs [48]. All the patients with MBC with HER2-positive tumor tissue in the current study were enrolled with progressive and/or recurrent disease.

There were no other significant differences in expression levels of the CTC-related genes between various breast cancer subtypes (estrogen receptor-positive, progesterone receptor positive, HER2-positive, triple-negative). However, some weak trends were observed (Figure 3c, sorted by CTC count, and Figure S4, hierarchical clustering by gene expression). High gene expression was generally associated with high CTC counts on the corresponding cytology slide. CTC gene expression in patients with triple-negative breast cancer was dispersed but tended to cluster with higher expression (Figure S4).

As described above, samples collected via a port had a much higher CTC count by cytology review. However, molecular profiling (Figure S5) showed only a slight trend for higher expression of CTC genes. Only the white blood cell marker *PTPRC* (CD45) was significantly higher in samples from patients with MBC collected via the port. These results suggest that the decrease in CTC counts by cytology review could be partially due to sample quality related to the slide preparation method since the morphology is lost to a greater extent compared to the gene expression. 

These results demonstrated that the cells harvested from the peripheral blood of patients with MBC by the Parsortix^®^ PC1 System could be analyzed with a qPCR method to evaluate the expression of genes using standard molecular techniques currently used in many clinical and/or research laboratory settings.

### 3.4. Combining Gene Expression Results with CTC Counts Reduces Classification Uncertainty 

Among the 176 samples evaluated for gene expression and CTC enumeration, there were no HVs that had both >1 observed CTC and expression of any CTC-related genes (excluding ERBB2) below a standard Ct threshold of 35.0. At the same time, 17 patients with MBC met both criteria (Figure S6, Node 6, far-right). Furthermore, only 15 of the 74 patients with MBC (20.3%) had both no observed CTCs and no expression of any CTC-related genes (Figure S6, Node 7, far-left). Additionally, 8 (10.6%) patients with MBC that had no CTCs detected by cytology were positive for at least 1 CTC-related gene, suggesting an increased sensitivity due to the inclusion of gene expression compared to the use of CTC counts alone.

### 3.5. RNA-Sequencing

**Figure 5:**
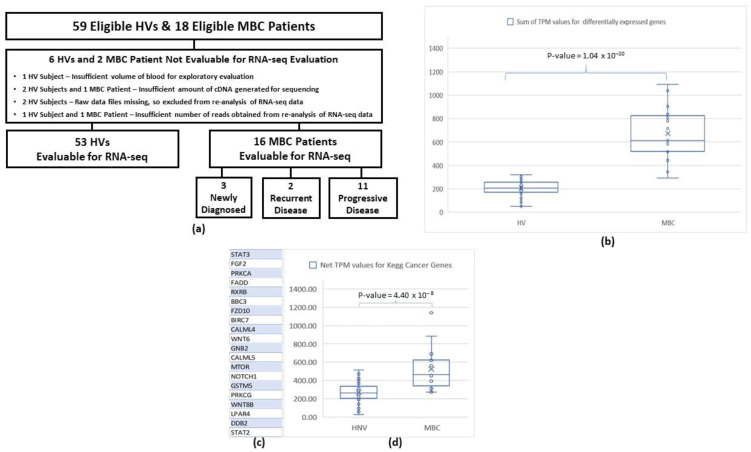
(**a**) Flow diagram for RNA-seq evaluation in eligible HV and MBC patients. (**b**) Sum of TPM values for all genes differentially expressed (*p* < 0.001) between Parsortix PC1 harvests obtained from HV comparators and MBC patients. (**c**) Genes from the KEGG Cancer Pathway were differentially expressed between the HV and MBC harvests (*p* < 0.05) (**d**). Net TPM score of these 20 genes for HV and MBC samples. CTC, circulating tumor cell; HV, healthy volunteer; MBC, metastatic breast cancer.

For unbiased evaluation of gene expression, RNA-seq was performed using cDNA prepared from RNA isolated from the CTC harvests of a subset of HVs and a small number of patients with MBC. Figure 5a provides the flow chart for the 18 eligible patients with MBC and 59 HVs whose second blood sample was intended to be used for the RNA-seq evaluation. A total of 53 HVs and 16 patients with MBC were evaluable for the analysis. The data contained a significant percentage of genomic DNA or other non-mRNA materials, representing approximately 90% of the sequenced reads. However, there were no significant differences (Student’s *t*-test) in the observed transcriptome reads, total reads, or percentage mapped to the transcriptome between the Parsortix^®^ PC1 System harvests obtained from the HVs and the patients with MBC. There was minimal non-human and ribosomal contamination. To assess the quality of the sequencing, a quality score (Q-score), which is a prediction of the probability of an error in base calling, was used. A high Q-score implies that a base call is more reliable and less likely to be incorrect, where Q = −10 log10 (e). For example, for base calls with a quality score of Q30, one base call in 1000 is predicted to be incorrect. The Q30 values for the sequencing data from the 53 HVs and 16 patients with MBC were all more than 90%, and the coverages (the rate of sequencing reads covering the reference genome cDNA sequence) were of a sufficient level in all samples. The transcriptome mapping results were consistent across all samples, so differential gene expression comparison was possible. 

TPM-normalized expression values per gene were generated for all samples. A total of 200 genes were found to be differentially expressed between HVs and patients with MBC with *p* < 0.005 (Student’s *t*-test), 424 genes were differentially expressed with *p* < 0.01, and 2570 genes were differentially expressed with *p* < 0.05. Figure 5b plots the sum of the TPM values from each sample for the differentially expressed genes between the HVs and patients with MBC with individual *p*-values of <0.001. The two groups are well differentiated by this set of genes, with a *p*-value of <0.0001 (Student’s *t*-test).

The expression of genes in the harvests from the Parsortix^®^ PC1 System known to be associated with the KEGG Pathways in cancer (https://www.kegg.jp, accessed on 20 October 2022) was examined for differential expression between the HVs and patients with MBC. Figure 5c lists the genes in the KEGG cancer pathways set that were determined to be differentially expressed between the HVs and patients with MBC with *p*-values of <0.05 (Student’s *t*-test). For illustrative purposes, a simple combination of the TPM values for these genes (net TPM score equals the sum of TPM for upregulated genes minus the sum of TPM values for downregulated genes) was calculated for each sample. Figure 5d illustrates the net TPM values derived from the 20 KEGG cancer pathway genes with *p*-values of <0.05 (Student’s *t*-test) for each of the HV and MBC samples. Significant discrimination was observed between the groups (Student’s *t*-test *p* < 0.0001). Since cancer signaling pathways were significantly enriched in the samples from the MBC patients compared to those from the HVs, this result suggests that the population of cells captured and harvested from the peripheral blood of patients with MBC processed by the Parsortix^®^ PC1 System does contain cancer cells.

Further examples are illustrated in Figures S7–S9, where harvests from various subgroups of patients with MBC are compared. Genes from the KEGG cancer pathway list were examined for differential expression between the identified groups. A net TPM score was generated for each sample using the genes that exhibited individual *p*-values of <0.05. Figure S7 lists the relevant genes and illustrates discrimination based on grouping patients by tissue HER2 status. Figure S8 provides a list of genes that appear to be associated with metastases to the lymph nodes. Figure S9 identifies a different gene expression profile potentially reflecting the presence of bone metastases.

The RNA-seq data confirm that the cells harvested by the Parsortix^®^ PC1 System from the peripheral blood of patients with MBC (and HVs) can be used to generate RNA-seq data that directly reflect cancer-associated gene expression patterns. This was demonstrated despite contaminating genomic DNA, illustrating the further potential to be realized with alternative RNA-seq sample preparation protocols.

### 3.6. HER2 FISH

HER2 is an important diagnostic component of breast cancer management and is typically evaluable at the single-cell level. Therefore, one of our exploratory evaluations involved interrogating the HER2 amplification status in the population of cells harvested from a subset of the HVs and patients with MBC using the Parsortix^®^ PC1 System.

**Figure 6:**
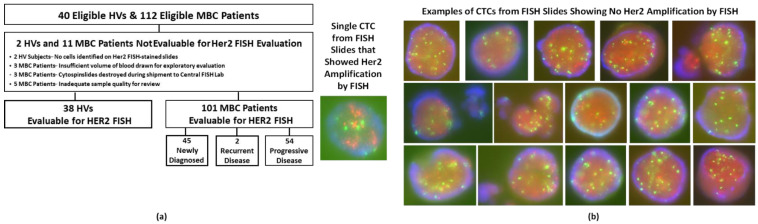
(**a**) Flow diagram for HER2 FISH evaluation in eligible MBC patients. (**b**) Example images of HER2 FISH-stained CTCs from MBC patients that were harvested by the Parsortix PC1 system and deposited onto cytology slides (images not to scale). CTC, circulating tumor cell; HV, healthy volunteer; MBC, metastatic breast cancer; FISH, fluorescence in situ hybridization.

Figure 6a provides the flow chart for the 40 eligible HVs and 112 patients with MBC, collected at The MD Anderson Cancer Center, University of Rochester Medical Center, and Northwestern University, whose second blood sample was intended to be used for the HER2 FISH evaluation. A total of 38 (95.0%) of the 40 eligible HVs and 101 (90.2%) of the 112 eligible patients with MBC had evaluable HER2 FISH-stained cytology slides; 5 (13.2%) of the 38 evaluable HVs and 28 (27.7%) of the 101 evaluable patients with MBC had one or more CTCs identified on their HER2 FISH-stained cytology slides (Table S2).

Figure 6b provides example images of the CTCs identified on the HER2 FISH slides of patients with MBC, including the single CTC from a newly diagnosed patient with HER2-positive MBC that demonstrated HER2 amplification. The single sample showing HER2 amplification represents 33.3% of the patients with MBC who had HER2-positive disease and had CTCs identified on their HER2 FISH slides (Table 5). Approximately 83% of the patients with MBC had estrogen receptor–positive and/or progesterone receptor–positive disease, and only 9.9% had HER2-positive breast cancer, which is lower than the proportion generally described in the literature (~20%). Therefore, although a reasonable number of samples were tested for HER2 status by FISH, only a minimal number were expected to have HER2 amplification. It should be noted that a strict definition of HER2 FISH positivity was used for this study, permitting resolution of all CTCs into either “HER2-amplified” or “HER2-not-amplified” status, as described previously in large cohorts of breast cancer patients and patients screened for entry to large clinical trials [31,32,33].

These results demonstrated that the Parsortix^®^ PC1 System can capture and harvest CTCs from the peripheral blood of patients with MBC and that the population of cells harvested can be effectively evaluated using a commercially available HER2 FISH assay and reagents.

## 4. Study Limitations

The open platform of the Parsortix system allows for multiple downstream analyses. However, this openness may limit immediate introduction to clinical environments as it requires additional technology, personnel, and equipment out of the box. It was difficult to comprehensively demonstrate all potential downstream evaluation methods that a user could employ to evaluate the cells harvested by the Parsortix^®^ PC1 System from the peripheral blood of patients with MBC. In this study, we focused on cytology evaluation, qPCR, FISH, and RNA-seq as examples of downstream analysis methods. These four methods cover a range of cytology and molecular techniques currently used in clinical laboratories and many FDA-cleared tests, including protein, RNA, and DNA analysis techniques.

We recognize that the study is limited by a lack of additional alternative downstream analyses, such as interrogation for mutations. Still, the study design and amount of testing that could be done for each subject were limited by the volume of blood that we could safely and ethically draw from the patients with MBC. In addition, the cytology evaluation method used to identify CTCs was based solely on morphological characteristics and, therefore, more subjective than an immunofluorescence staining assay. Additionally, the slide preparation method for the cytology and FISH analyses proved to have a high cell loss, with 37–51% of the harvested cells not being deposited on the slides. This most likely resulted in underestimation of the number of CTCs in the blood samples. However, a correction factor would not be practical for the high number of samples with no CTCs observed, as the cell loss caused by the cytology slide preparation method was highly variable. However, we used cytocentrifugation (also known as cytospin) because it is a routine procedure performed in pathology laboratory practice and readily available to most laboratories. In addition to the cell loss caused by the cytology slide preparation method, the degree of subjectivity around the cytomorphological interpretation/identification of CTCs is another study limitation. CTC-specific markers (such as pan-cytokeratin), in addition to the Wright-Giemsa staining, to assess the phenotype of the atypical, non-normal, and malignant cells identified on the CTC slides may have provided additional evidence that the malignant cells indeed were CTCs. However, the morphological scoring of CTCs has more clinical value and avoids the use of potentially poor-performing antibodies. Future efforts to establish diagnostic criteria for morphologic CTC evaluation are needed in conjunction with broader adoption of the Parsortix^®^ PC1 System in clinical practice.

The study specified that a minimum volume of blood needed to be available (≥5 mL) for the processing of each sample instead of specifying that an exact volume of blood be used for each sample (e.g., 7.5 mL or 8 mL). However, the use of varying volumes of blood for each sample makes it impossible to directly compare results between samples that used different volumes of blood. Furthermore, there is known variability in the numbers of cells present between different tubes of blood taken from the same patient (tube-to-tube variability).

No FDA-cleared orthogonal method was tested to demonstrate the equivalency of the Parsortix^®^ PC1 System for the capture and harvest of CTCs. The FDA-cleared CELLSEARCH^®^ System was not deemed suitable for inclusion as a reference method because its technological operating characteristics are fundamentally different. The CELLSEARCH^®^ System enriches CTCs based on immunoaffinity to EpCAM, magnetically immobilizes the cells inside a visualization chamber, stains the captured cells with DAPI and anti-EpCAM and anti-CD45 antibodies, captures fluorescent images of the stained cells, and presents the digitized images to a user for identification of CTCs. In contrast, the Parsortix^®^ PC1 System uses microfluidics to enrich cells based on their size and deformability, and it allows the cells captured by the microfluidic device to be harvested into a small buffer volume for further evaluation. The Parsortix PC1 System may have an advantage over the CELL-SEARCH System in detecting mesenchymal CTCs, but CTCs can be epithelial, mesenchymal, or hybrid. Furthermore, the clinical significance of the amount and type of CTC on therapeutic efficacy in MBC as well as primary breast cancer is still unknown. It remains to be seen how the CTCs detected in the current study will affect the clinical outcome of MBC patients.

Follow-up studies will be required to demonstrate the clinical utility of CTC enrichment in patients with MBC using the Parsortix^®^ PC1 System in combination with analytically validated subsequent downstream analysis methods for molecular characteristics. This study was designed only to test the enrichment platform, and thus, the samples obtained were de-identified, and no follow-up clinical data was collected.

Overall, the results of this study showed that the population of cells harvested by the Parsortix^®^ PC1 System from the peripheral blood of patients with MBC could be evaluated using currently available laboratory methods for the identification and characterization of CTCs. As CTCs are obtained from peripheral blood (i.e., a liquid biopsy), additional samples can easily be obtained with minimal impact if there is a processing error and/or no CTCs are present in the population of cells harvested or if additional blood volume is deemed necessary to meet downstream assay performance requirements.

## 5. Conclusions

The HOMING Study “Harvest of CTCs from Patients with MBC using the Parsortix^®^ System” was a multi-center, prospective, blinded study that enrolled over 200 evaluable healthy volunteers and 200 patients with MBC at four US-based clinical sites to demonstrate the ability of the system to enrich CTC for subsequent downstream analysis. The data here showed that cells harvested from the peripheral blood of the eligible HVs and patients with MBC using the Parsortix^®^ PC1 System can be successfully evaluated using cytology (i.e., Wright-Giemsa staining), qRT-PCR, RNA-sequencing, and FISH. The data generated from this study were used to support a De Novo request for classification of the Parsortix^®^ PC1 system (DEN200062) as a Class II prescription device that was granted by the FDA on 24 May 2022 (https://www.accessdata.fda.gov/cdrh_docs/pdf20/DEN200062.pdf, accessed on 20 October 2022). 

## Figures and Tables

**Table 1:** Summary of eligible MBC patients and HVs demographics and clinical characteristics.

Parameter and Categories	Eligible HV Subjects with Evaluable Results	Eligible MBC Patients with Evaluable Results	Eligible MBC Patients with Evaluable Results		
			Newly Diagnosed	Progression /Recurring	
No. of eligible participants	204	207	74 (35.7%)	133 (64.3%)	
Age at the time of the blood collection					
<57 years old	170 (83.3%)	103 (49.8%)	37 (50.0%)	66 (49.6%)	
≥57 years old	34 (16.7%)	104 (50.2%)	37 (50.0%)	67 (50.4%)	
Fisher exact *p*-value	<0.001		1.000		
Blood collection method					
Venipuncture	204 (100.0%)	171 (82.6%)	70 (94.6%)	101 (75.9%)	
Port	0 (0.0%)	36 (17.4%)	4 (5.4%)	32 (24.1%)	
Fisher exact *p*-value	<0.001		<0.001		
Menopausal status					
Pre-menopausal	130 (63.7%)	36 (17.4%)	15 (20.3%)	21 (15.8%)	
Post-menopausal	53 (26.0%)	158 (76.3%)	50 (67.6%)	108 (81.2%)	
Unknown	21 (10.3%)	13 (6.3%)	9 (12.2%)	4 (3.0%)	
Fisher exact *p*-value *	<0.001		0.328		
Race/ethnicity					
White	109 (53.4%)	151 (72.9%)	55 (74.3%)	96 (72.2%)	
Black	20 (9.8%)	22 (10.6%)	8 (10.8%)	14 (10.5%)	
Hispanic	40 (19.6%)	21 (10.1%)	8 (10.8%)	13 (9.8%)	
Other/unknown	35 (17.2%)	13 (6.3%)	3 (4.1%)	10 (7.5%)	
Fisher exact *p*-value	<0.001		0.826		
Previous history of cancer?					
Yes	0 (0.0%)	15 (7.2%)	5 (6.8%)	10 (7.5%)	
No	204 (100.0%)	192 (92.8%)	69 (93.2%)	123 (92.5%)	
Fisher exact *p*-value	<0.001		1.000		
Breast cancer ER status					
Positive	---	160 (77.3%)	55 (74.3%)	105 (78.9%)	
Negative	---	45 (21.7%)	18 (24.3%)	27 (20.3%)	
Unknown	---	2 (1.0%)	1 (1.4%)	1 (0.8%)	
Fisher exact *p*-value *	---		0.487		
Breast cancer PR status					
Positive	---	127 (61.4%)	44 (59.5%)	83 (62.4%)	
Negative	---	73 (35.3%)	27 (36.5%)	46 (34.6%)	
Unknown	---	7 (3.4%)	3 (4.1%)	4 (3.0%)	
Fisher exact *p*-value *	---		0.761		
Breast cancer HR status					
Positive	---	39 (79.7%)	57 (77.0%)	108 (81.2%)	
Negative	---	165 (18.8%)	16 (21.6%)	23 (17.3%)	
Unknown	---	3 (1.4%)	1 (1.4%)	2 (1.5%)	
Fisher exact *p*-value *	---		0.448		
Breast cancer HER2-Neu status					
Negative	---	165 (79.7%)	66 (89.2%)	99 (74.4%)	
Positive	---	27 (13.0%)	4 (5.4%)	23 (17.3%)	
Equivocal	---	8 (3.9%)	1 (1.4%)	7 (5.3%)	
Unknown	---	7 (3.4%)	3 (4.1%)	4 (3.0%)	
Fisher exact *p*-value *	---		0.014		
Breast cancer TNBC status					
TNBC	---	33 (15.9%)	54 (50.5%)	98 (73.7%)	
Non-TNBC	---	152 (73.4%)	13 (12.1%)	20 (15.0%)	
Unknown	---	22 (10.6%)	7 (6.5%)	15 (11.3%)	
Fisher exact *p*-value *	---		0.675		
Metastatic disease status determined by (more than one may apply)					Fisher exact *p*-value **
Imaging	---	198 (95.7%)	70 (94.6%)	128 (96.2%)	0.724
Rising tumor markers	---	5 (2.4%)	2 (2.7%)	3 (2.3%)	1.000
Physical signs and symptoms	---	17 (8.2%)	8 (10.8%)	9 (6.8%)	0.306
Physician determination	---	4 (1.9%)	1 (1.4%)	3 (2.3%)	1.000
Other (primarily biopsy)	---	62 (30.0%)	49 (66.2%)	13 (9.8%)	0.000
Sites of metastasis (more than one may apply)					Fisher exact *p*-value **
Abdomen	---	6 (2.9%)	3 (4.1%)	3 (2.3%)	0.669
Adrenal gland	---	3 (1.4%)	0 (0.0%)	3 (2.3%)	0.554
Ascites	---	1 (0.5%)	1 (1.4%)	0 (0.0%)	0.357
Bone	---	139 (67.1%)	42 (56.8%)	97 (72.9%)	0.021
Brain	---	29 (14%)	5 (6.8%)	24 (18.0%)	0.035
Chest wall	---	16 (7.7%)	4 (5.4%)	12 (9.0%)	0.425
Liver	---	80 (38.6%)	10 (13.5%)	70 (52.6%)	0.000
Lung	---	77 (37.2%)	23 (31.1%)	54 (40.6%)	0.181
Lymph nodes	---	107 (51.7%)	33 (44.6%)	74 (55.6%)	0.148
Other site(s)	---	33 (15.9%)	9 (12.2%)	24 (18.0%)	0.325

* Comparisons do not include the “Unknown” category. MBC, metastatic breast cancer; HV, healthy volunteer; TNBC, triple-negative breast cancer; ER, estrogen receptor; PR, progesterone receptor; HR, hormone receptor. ** Fisher exact *p*-value for comparison between MBC patients with newly diagnosed disease and those with progression/recurring disease.

**Table 2:** CTC prevalence rates from initial cytopathology review in MBC patients and HVs by demographic and clinical characteristics.

Parameter and Categories	Eligible HV Subjects and MBC Patients with Evaluable Cytology Slides											
	All Eligible and Evaluable HV Subjects			All Eligible and Evaluable MBC Patients			Newly Diagnosed MBC Patients			Progression/Recurring MBC Patients		
	N	≥1 CTC	≥5 CTC	N	≥1 CTC	≥5 CTC	N	≥1 CTC	≥5 CTC	N	≥1 CTC	≥5 CTC
All participants	192	19 (9.9%)	2 (1.0%)	194	94 (48.5%)	44 (22.7%)	69	23 (33.3%)	9 (13.0%)	125	71 (56.8%)	35 (28.0%)
Age at the time of the blood collection												
<57 Years Old	159	15 (9.4%)	1 (0.6%)	96	41 (42.7%)	17 (17.7%)	33	6 (18.2%)	3 (9.1%)	63	35 (55.6%)	14 (22.2%)
≥57 Years Old	33	4 (12.1%)	1 (3.0%)	98	53 (54.1%)	27 (27.6%)	36	17 (47.2%)	6 (16.7%)	62	36 (58.1%)	21 (33.9%)
Fisher exact test *p*-value		0.748	0.315		0.117	0.123		0.012	0.481		0.857	0.167
Blood collection method												
via Venipuncture	192	19 (9.9%)	2 (1.0%)	159	61 (38.4%)	22 (13.8%)	65	19 (29.2%)	7 (10.8%)	94	42 (44.7%)	15 (16.0%)
via Port	0	0 (0.0%)	0 (0.0%)	35	33 (94.3%)	22 (62.9%)	4	4 (100.0%)	2 (50.0%)	31	29 (93.5%)	20 (64.5%)
Fisher exact test *p*-value		---	---		<0.001	<0.001		0.010	0.080		<0.001	<0.001
Menopausal status												
Pre-Menopausal	124	12 (9.7%)	1 (0.8%)	34	14 (41.2%)	7 (20.6%)	14	2 (14.3%)	1 (7.1%)	20	12 (60.0%)	6 (30.0%)
Post-Menopausal	51	5 (9.8%)	1 (2.0%)	147	75 (51.0%)	35 (23.8%)	46	19 (41.3%)	8 (17.4%)	101	56 (55.4%)	27 (26.7%)
Unknown	17	2 (11.8%)	0 (0.0%)	13	5 (38.5%)	2 (15.4%)	9	2 (22.2%)	0 (0.0%)	4	3 (75.0%)	2 (50.0%)
Fisher exact test *p*-value		1.000	0.499		0.344	0.832		0.108	0.671		0.808	0.787
Race/ethnicity												
White	102	7 (6.9%)	1 (1.0%)	141	71 (50.4%)	31 (22.0%)	51	19 (37.3%)	7 (13.7%)	90	52 (57.8%)	24 (26.7%)
Black	20	5 (25.0%)	0 (0.0%)	21	13 (61.9%)	9 (42.9%)	7	2 (28.6%)	2 (28.6%)	14	11 (78.6%)	7 (50.0%)
Hispanic	37	4 (10.8%)	1 (2.7%)	21	4 (19.0%)	1 (4.8%)	8	2 (25.0%)	0 (0.0%)	13	2 (15.4%)	1 (7.7%)
Other/Unknown	33	3 (9.1%)	0 (0.0%)	11	6 (54.5%)	3 (27.3%)	3	0 (0.0%)	0 (0.0%)	8	6 (75.0%)	3 (37.5%)
Fisher exact test *p*-value		0.114	0.719		0.022	0.026		0.740	0.388		0.004	0.084
Breast cancer ER status												
Positive	0	0 (0.0%)	0 (0.0%)	151	70 (46.4%)	35 (23.2%)	52	18 (34.6%)	7 (13.5%)	99	52 (52.5%)	28 (28.3%)
Negative	0	0 (0.0%)	0 (0.0%)	42	24 (57.1%)	9 (21.4%)	16	5 (31.3%)	2 (12.5%)	26	19 (73.1%)	7 (26.9%)
Fisher’s Exact Test *p*-value		---	---		0.227	1.000		1.000	1.000		0.076	1.000
Breast Cancer PR Status												
Positive	0	0 (0.0%)	0 (0.0%)	121	57 (47.1%)	26 (21.5%)	42	13 (31.0%)	6 (14.3%)	79	44 (55.7%)	20 (25.3%)
Negative	0	0 (0.0%)	0 (0.0%)	67	33 (49.3%)	15 (22.4%)	24	9 (37.5%)	3 (12.5%)	43	24 (55.8%)	12 (27.9%)
Unknown	0	0 (0.0%)	0 (0.0%)	6	4 (66.7%)	3 (50.0%)	3	1 (33.3%)	0 (0.0%)	3	3 (100.0%)	3 (100.0%)
Fisher exact test *p*-value		---	---		0.879	1.000		0.599	1.000		1.000	0.830
Breast cancer HER2-Neu status												
Negative	0	0 (0.0%)	0 (0.0%)	156	70 (44.9%)	33 (21.2%)	62	21 (33.9%)	8 (12.9%)	94	49 (52.1%)	25 (26.6%)
Positive	0	0 (0.0%)	0 (0.0%)	24	17 (70.8%)	8 (33.3%)	3	1 (33.3%)	1 (33.3%)	21	16 (76.2%)	7 (33.3%)
Equivocal	0	0 (0.0%)	0 (0.0%)	8	4 (50.0%)	2 (25.0%)	1	1 (100.0%)	0 (0.0%)	7	3 (42.9%)	2 (28.6%)
Unknown	0	0 (0.0%)	0 (0.0%)	6	3 (50.0%)	1 (16.7%)	3	0 (0.0%)	0 (0.0%)	3	3 (100.0%)	1 (33.3%)
Fisher exact test *p*-value		---	---		0.061	0.378		0.533	0.452		0.098	0.809
Breast cancer TNBC status												
Non-TNBC	0	0 (0.0%)	0 (0.0%)	143	66 (46.2%)	33 (23.1%)	51	17 (33.3%)	7 (13.7%)	92	49 (64.1%)	26 (28.3%)
TNBC	0	0 (0.0%)	0 (0.0%)	31	19 (61.3%)	6 (19.4%)	12	5 (4.2%)	2 (16.7%)	19	14 (73.7%)	4 (21.0%)
Unknown	0	0 (0.0%)	0 (0.0%)	20	9 (45.0%)	5 (25.0%)	6	1 (16.7%)	0 (0.0%)	14	8 (57.1%)	5 (35.7%)
Fisher exact test *p*-value		---	---		0.4499	0.6523		0.5859	0.1019		0.6209	0.5195
Sites of metastasis (more than one may apply)												
Abdomen	0	0 (0.0%)	0 (0.0%)	6	3 (50.0%)	2 (33.3%)	3	1 (33.3%)	1 (33.3%)	3	2 (66.7%)	1 (33.3%)
Adrenal gland	0	0 (0.0%)	0 (0.0%)	3	2 (66.7%)	1 (33.3%)	0	0 (0.0%)	0 (0.0%)	3	2 (66.7%)	1 (33.3%)
Ascites	0	0 (0.0%)	0 (0.0%)	1	0 (0.0%)	0 (0.0%)	1	0 (0.0%)	0 (0.0%)	0	0 (0.0%)	0 (0.0%)
Bone	0	0 (0.0%)	0 (0.0%)	132	72 (54.5%)	40 (30.3%)	39	14 (35.9%)	9 (23.1%)	93	58 (62.4%)	31 (33.3%)
Brain	0	0 (0.0%)	0 (0.0%)	26	17 (65.4%)	9 (34.6%)	4	2 (50.0%)	1 (25.0%)	22	15 (68.2%)	8 (36.4%)
Chest wall	0	0 (0.0%)	0 (0.0%)	16	11 (68.8%)	4 (25.0%)	4	3 (75.0%)	2 (50.0%)	12	8 (66.7%)	2 (16.7%)
Kidney	0	0 (0.0%)	0 (0.0%)	0	0 (0.0%)	0 (0.0%)	0	0 (0.0%)	0 (0.0%)	0	0 (0.0%)	0 (0.0%)
Liver	0	0 (0.0%)	0 (0.0%)	73	41 (56.2%)	19 (26.0%)	9	2 (22.2%)	2 (22.2%)	64	39 (60.9%)	17 (26.6%)
Lung	0	0 (0.0%)	0 (0.0%)	70	35 (50.0%)	19 (27.1%)	22	6 (27.3%)	1 (4.5%)	48	29 (60.4%)	18 (37.5%)
Lymph nodes	0	0 (0.0%)	0 (0.0%)	101	48 (47.5%)	19 (18.8%)	31	10 (32.3%)	3 (9.7%)	70	38 (54.3%)	16 (22.9%)
Other site(s)	0	0 (0.0%)	0 (0.0%)	33	15 (45.5%)	4 (12.1%)	9	4 (44.4%)	1 (11.1%)	24	11 (45.8%)	3 (12.5%)

MBC, metastatic breast cancer; HV, healthy volunteer; TNBC, triple-negative breast cancer; ER, estrogen receptor; PR, progesterone receptor; HR, hormone receptor.

**Table 3:** Summary of CTCs from review of evaluable Wright-Giemsa stained cytology slides from 192 HV subjects and 194 MBC patients.

No. of CTCs Observed	Cytopathology Review Results: Eligible HV Subjects and MBC Patients with Adequate Cytology Slides (% [95% CI])					
	Evaluable HV Subjects	Evaluable MBC Patients	Fisher Exact *p*-Value	Newly Diagnosed MBC Patients	Recurring/Progressing MBC Patients	Fisher Exact *p*-Value
0 CTC	173 (90.1% [85.1–93.6%])	100 (51.5% [44.6–58.5%])	---	46 (66.7% [54.9–76.6%])	54 (43.2% [34.8–52.0%])	---
≥1 CTC	19 (9.9% [6.4–14.9%])	94 (48.5% [41.5–55.4%])	<0.001	23 (33.3% [23.4–45.1%])	71 (56.8% [48.0–65.2%])	0.003
≥2 CTC	6 (3.1% [1.4–6.6%])	77 (39.7% [33.1–46.7%])	<0.001	18 (26.1% [17.2–37.5%])	59 (47.2% [38.7–55.9%])	0.006
≥3 CTC	4 (2.1% [0.8–5.2%])	63 (32.5% [26.3–39.4%])	<0.001	14 (20.3% [12.5–31.2%])	49 (39.2% [31.1–48.0%])	0.010
≥4 CTC	2 (1.0% [0.3–3.7%])	53 (27.3% [21.5–34.0%])	<0.001	12 (17.4% [10.2–28.0%])	41 (32.8% [25.2–41.4%])	0.028
≥5 CTC	2 (1.0% [0.3–3.7%])	44 (22.7% [17.3–29.1%])	<0.001	9 (13.0% [7.0–23.0%])	35 (28.0% [20.9–36.4%])	0.020
≥10 CTC	1 (0.5% [0.1–2.9%])	30 (15.5% [11.0–21.2%])	<0.001	4 (5.8% [2.3–14.0%])	26 (20.8% [14.6–28.7%])	0.006
TOTAL N	192	194		69	125	

HV, healthy volunteer; MBC, metastatic breast cancer.

**Table 4:** Proportions of HV subjects and MBC patients with positive gene expression using a Ct value threshold of ≤35.0 for each gene to determine positivity and comparisons to numbers of CTCs observed on Wright-Giemsa-stained slides during cytopathology review.

Group *	N	GAPDH	B2M	GYPA	PTPRC	KRT19	EpCAM	ERBB2	TWIST1	SNAI2	KRT19, EpCAM, ERBB2, TWIST &/or SNAI2	KRT19, EpCAM, TWIST &/or SNAI2
Negative Controls	18	0.0%	0.0%	0.0%	0.0%	0.0%	0.0%	0.0%	0.0%	0.0%	0.0%	0.0%
Positive Controls	18	100.0%	100.0%	5.6%	16.7%	100.0%	100.0%	100.0%	100.0%	100.0%	100.0%	100.0%
All HVs	102	99.0%	100.0%	2.0%	100.0%	0.0%	1.0%	19.6%	1.0%	0.0%	19.6%	1.0%
with a CTC count	99 (97.1%)	100.0%	100.0%	2.0%	100.0%	0.0%	1.0%	20.2%	1.0%	0.0%	20.2%	1.0%
with 0 CTC	83 (83.8%)	100.0%	100.0%	2.4%	100.0%	0.0%	1.2%	21.7%	1.2%	0.0%	21.7%	1.2%
with 1 CTC	11 (11.1%)	100.0%	100.0%	0.0%	100.0%	0.0%	0.0%	18.2%	0.0%	0.0%	18.2%	0.0%
with 2–4 CTCs	3 (3.1%)	100.0%	100.0%	0.0%	100.0%	0.0%	0.0%	0.0%	0.0%	0.0%	0.0%	0.0%
with 5–9 CTCs	1 (1.0%)	100.0%	100.0%	0.0%	100.0%	0.0%	0.0%	0.0%	0.0%	0.0%	0.0%	0.0%
with ≥10 CTCs	1 (1.0%)	100.0%	100.0%	0.0%	100.0%	0.0%	0.0%	0.0%	0.0%	0.0%	0.0%	0.0%
All MBC patients	74	98.6%	100.0%	5.4%	100.0%	21.6%	13.5%	47.3%	5.4%	0.0%	52.7%	24.3%
with a CTC count	71 (95.9%)	98.6%	100.0%	5.6%	100.0%	22.5%	14.1%	47.9%	5.6%	0.0%	53.5%	25.4%
with 0 CTC	31 (43.7%)	100.0%	100.0%	3.2%	100.0%	22.6%	9.7%	48.4%	6.5%	0.0%	54.8%	25.8%
with 1 CTC	10 (14.1%)	100.0%	100.0%	0.0%	100.0%	0.0%	0.0%	30.0%	0.0%	0.0%	30.0%	0.0%
with 2–4 CTCs	14 (19.7%)	92.9%	100.0%	0.0%	100.0%	28.6%	21.4%	42.9%	7.1%	0.0%	57.1%	35.7%
with 5–9 CTCs	5 (7.0%)	100.0%	100.0%	0.0%	100.0%	0.0%	0.0%	40.0%	0.0%	0.0%	40.0%	0.0%
with ≥10 CTCs	11 (15.5%)	100.0%	100.0%	27.3%	100.0%	45.5%	36.4%	72.7%	9.1%	0.0%	72.7%	45.5%
All newly diagnosed MBC patients	21	100.0%	100.0%	9.5%	100.0%	23.8%	9.5%	66.7%	14.3%	0.0%	71.4%	28.6%
with a CTC count	20 (95.2%)	100.0%	100.0%	10.0%	100.0%	25.0%	10.0%	65.0%	15.0%	0.0%	70.0%	30.0%
with 0 CTC	12 (60.0%)	100.0%	100.0%	8.3%	100.0%	33.3%	8.3%	66.7%	16.7%	0.0%	75.0%	41.7%
with 1 CTC	2 (10.0%)	100.0%	100.0%	0.0%	100.0%	0.0%	0.0%	50.0%	0.0%	0.0%	50.0%	0.0%
with 2–4 CTCs	3 (15.0%)	100.0%	100.0%	0.0%	100.0%	0.0%	0.0%	66.7%	0.0%	0.0%	66.7%	0.0%
with 5–9 CTCs	2 (10.0%)	100.0%	100.0%	0.0%	100.0%	0.0%	0.0%	50.0%	0.0%	0.0%	50.0%	0.0%
with ≥10 CTCs	1 (5.0%)	100.0%	100.0%	100.0%	100.0%	100.0%	100.0%	100.0%	100.0%	0.0%	100.0%	100.0%
All recurrent/progressive MBC patients	53	98.1%	100.0%	3.8%	100.0%	20.8%	15.1%	39.6%	1.9%	0.0%	45.3%	22.6%
with CTC count	51 (96.2%)	98.0%	100.0%	3.9%	100.0%	21.6%	15.7%	41.2%	2.0%	0.0%	47.1%	23.5%
with 0 CTC	19 (37.2%)	100.0%	100.0%	0.0%	100.0%	15.8%	10.5%	36.8%	0.0%	0.0%	42.1%	15.8%
with 1 CTC	8 (15.7%)	100.0%	100.0%	0.0%	100.0%	0.0%	0.0%	25.0%	0.0%	0.0%	25.0%	0.0%
with 2–4 CTCs	11 (21.6%)	90.9%	100.0%	0.0%	100.0%	36.4%	27.3%	36.4%	9.1%	0.0%	54.5%	45.5%
with 5–9 CTCs	3 (5.9%)	100.0%	100.0%	0.0%	100.0%	0.0%	0.0%	33.3%	0.0%	0.0%	33.3%	0.0%
with ≥10 CTCs	10 (19.6%)	100.0%	100.0%	20.0%	100.0%	40.0%	30.0%	70.0%	0.0%	0.0%	70.0%	40.0%

HV, healthy volunteer; MBC, metastatic breast cancer. * CTC count and gene expression are from separate, parallel blood tubes.

**Table 5:** Results from evaluation of Parsortix harvests using HER2 FISH. The number and proportion of samples with CTCs identified on the FISH slide and the number and proportion of samples showing CTCs with HER2 amplification in the HV subjects and MBC patients by their tissue HER2 status.

Parameter and Categories	HVs	MBC Patients by Tissue HER2 Status			
		Unknown	Equivocal	Negative	Positive
n	38	3	4	84	10
No. with CTC identified	5 (13.2%)	0 (0.0%)	2 (50.0%)	23 (27.4%)	3 (30.0%)
No. with HER2 amplified in CTC	0 of 5 (0%)	-	0 of 2 (0%)	0 of 23 (0%)	1 of 3 (33.3%)

FISH, fluorescence in situ hybridization; HV, healthy volunteer; MBC, metastatic breast cancer.

## Data Availability

The data can be shared up on request.

## Supplementary Materials

The following supporting information can be downloaded at: https://www.mdpi.com/article/10.3390/cancers14215238/s1, Figure S1: Comparison of linearity, Figure S2: Gene expression in samples with and without detected CTCs in corresponding cytology slides, Figure S3: CTC-related gene expression in newly diagnosed and recurrent/progressive metastatic breast cancer (MBC), Figure S4: CTC count and CTC-related gene expression hierarchical clustering, Figure S5: Trend for higher CTC-related gene expression from samples collected from a port, Figure S6: Decision Tree, Figure S7: Genes from the KEGG Cancer for HER Primary Tumors, Figure S8: Genes from the KEGG Cancer for LN Positive MBC, Figure S9: Genes from the KEGG Cancer for Bone Metastasis MBC, Table S1: Proportions of healthy volunteers and MBC patients with positive gene expression, Table S2: Summary of CTC counts on HER2.
