# Supplementary material for: A Multi-Center Clinical Study to Harvest and Characterize Circulating Tumor Cells from Patients with Metastatic Breast Cancer Using the Parsortix® PC1 System
Source: Cancers (Basel). 2022 Oct 26;14(21):5238. doi: 10.3390/cancers14215238 (PMC9656921; doi:10.3390/cancers14215238)
Supplement: Supplementary file 1 [file cancers-14-05238-s001.zip › cancers-1960584-supplementary.pdf]

Figure S1. Comparison of linearity using live, SKBR3, MCF7, and Hs 578T cells spiked into 7.5 mL of blood over a range of 2 to ~100 cells and processed using Parsortix PC1 Systems where captured cells were harvested and deposited onto CytoSpin slides for identification using Wright-Giemsa staining (a) or pre-labeled with a fluorescent dye prior to spiking and harvested directly into 96-well plates for enumeration (b).

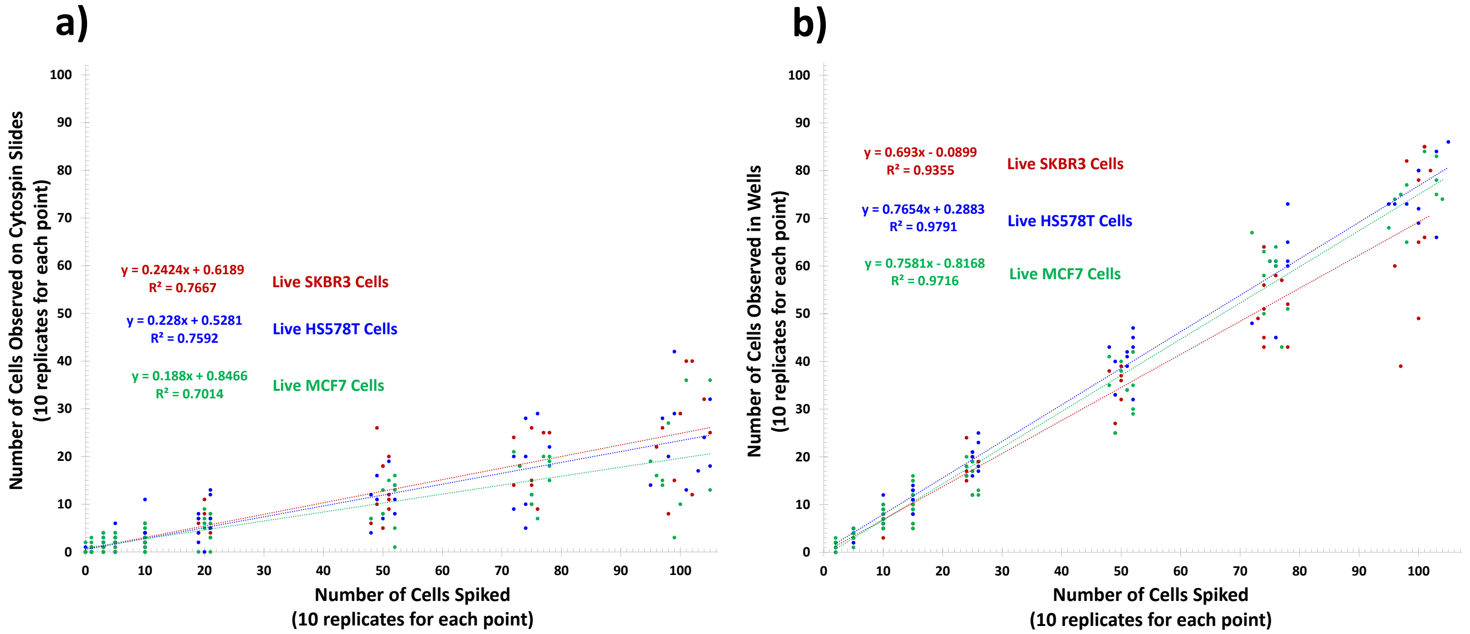

An evaluation of the cytocentrifugation method used for the preparation of the cytology slides in this study was conducted to assess the efficiency (i.e., cell retention) and linearity of the method. This analytical evaluation used 7.5-mL aliquots of blood collected from healthy women that were spiked with known numbers of live cultured tumor cells (between 0 and ~100 SKBR3, MCF7 or Hs 578T cells). The spiked blood samples were processed on Parsortix PC1 systems, and the captured cells were harvested and deposited onto charged glass cytology slides (Cytospin slides) using the cytocentrifugation method. The cells on the slides were fixed, Wright-Giemsa stained, and identified using light microscopy. The results showed that significant cell loss occurred for all three of the cell lines evaluated as a result of the cytocentrifugation method used for the preparation of the cytology slides and/or the Wright-Giemsa staining procedure (**Figure S1**). The average percentages of SKBR3, MCF7, and Hs 578T cells observed on the Wright-Giemsa-stained cytology slides over all of the spiking levels were 31% (median = 28%), 36% (median = 25%) and 28% (median = 23%), respectively. In contrast, when the captured cells were harvested directly into 96-well plates, the average percentages of pre-labeled SKBR3, MCF7, and Hs 578T cells observed in the wells were 69% (median = 70%), 73% (median = 76%), and 79% (median = 80%), respectively (**Figure S1**). These results indicated that 37%-51% of the cells harvested by the Parsortix PC1 system were lost due to the cytology slide preparation method and/or Wright-Giemsa staining procedure.

Table S1. Proportions of healthy volunteers and MBC patients with positive gene expression according to gene-specific Ct value thresholds to determine positivity (≤ threshold indicates positivity) and comparisons to numbers of CTCs observed on Wright-Giemsa stained slides during cytopathology review. *Percentages of CTC count groups are out of the number of patients in the group with a CTC count available.

Gene-specific Ct value threshold (≤ threshold = positive)		35.0				35.6	34.5	34.1	36.0	39.5		
Group	N	GAPDH	B2M	GYPA	PTPRC	KRT19	EpCAM	ERBB2	TWIST1	SNAI2	KRT19, EpCAM, ERBB2, TWIST and/or SNAI2	KRT19, EpCAM, TWIST and/or SNAI2
Negative controls	18	0.0%	0.0%	0.0%	0.0%	0.0%	0.0%	0.0%	0.0%	0.0%	0.0%	0.0%
Positive controls	18	100.0%	100.0%	5.6%	16.7%	100.0%	100.0%	100.0%	100.0%	100.0%	100.0%	100.0%
All healthy volunteers	102	99.0%	100.0%	2.0%	100.0%	0.0%	0.0%	3.9%	4.9%	0.0%	8.8%	4.9%
- with a CTC count *	99 (97.1%)	100.0%	100.0%	2.0%	100.0%	0.0%	0.0%	4.0%	5.1%	0.0%	9.1%	5.1%
- with 0 CTCs *	83 (83.8%)	100.0%	100.0%	2.4%	100.0%	0.0%	0.0%	4.8%	6.0%	0.0%	10.8%	6.0%
- with 1 CTC *	11 (11.1%)	100.0%	100.0%	0.0%	100.0%	0.0%	0.0%	0.0%	0.0%	0.0%	0.0%	0.0%
- with 2–4 CTCs *	3 (3.1%)	100.0%	100.0%	0.0%	100.0%	0.0%	0.0%	0.0%	0.0%	0.0%	0.0%	0.0%
- with 5–9 CTCs *	1 (1.0%)	100.0%	100.0%	0.0%	100.0%	0.0%	0.0%	0.0%	0.0%	0.0%	0.0%	0.0%
- with ≥10 CTCs *	1 (1.0%)	100.0%	100.0%	0.0%	100.0%	0.0%	0.0%	0.0%	0.0%	0.0%	0.0%	0.0%
All MBC patients	74	98.6%	100.0%	5.4%	100.0%	24.3%	10.8%	25.7%	9.5%	10.8%	41.9%	29.7%
- with a CTC count *	71 (95.9%)	98.6%	100.0%	5.6%	100.0%	25.4%	11.3%	25.4%	9.9%	11.3%	42.3%	31.0%
- with 0 CTCs *	31 (43.7%)	100.0%	100.0%	3.2%	100.0%	25.8%	6.5%	19.4%	9.7%	9.7%	38.7%	32.3%
- with 1 CTC *	10 (14.1%)	100.0%	100.0%	0.0%	100.0%	0.0%	0.0%	20.0%	0.0%	0.0%	20.0%	0.0%
- with 2–4 CTCs *	14 (19.7%)	92.9%	100.0%	0.0%	100.0%	28.6%	21.4%	21.4%	7.1%	7.1%	50.0%	35.7%
- with 5–9 CTCs *	5 (7.0%)	100.0%	100.0%	0.0%	100.0%	0.0%	0.0%	20.0%	0.0%	0.0%	20.0%	0.0%
- with ≥10 CTCs *	11 (15.5%)	100.0%	100.0%	27.3%	100.0%	54.5%	27.3%	54.5%	27.3%	36.4%	72.7%	63.6%
All newly diagnosed MBC patients	21	100.0%	100.0%	9.5%	100.0%	23.8%	9.5%	38.1%	14.3%	19.0%	52.4%	28.6%
- with a CTC count *	20 (95.2%)	100.0%	100.0%	10.0%	100.0%	25.0%	10.0%	35.0%	15.0%	20.0%	50.0%	30.0%
- with 0 CTCs *	12 (60.0%)	100.0%	100.0%	8.3%	100.0%	33.3%	8.3%	33.3%	16.7%	25.0%	58.3%	41.7%
- with 1 CTC *	2 (10.0%)	100.0%	100.0%	0.0%	100.0%	0.0%	0.0%	0.0%	0.0%	0.0%	0.0%	0.0%
- with 2–4 CTCs *	3 (15.0%)	100.0%	100.0%	0.0%	100.0%	0.0%	0.0%	66.7%	0.0%	0.0%	66.7%	0.0%
- with 5–9 CTCs *	2 (10.0%)	100.0%	100.0%	0.0%	100.0%	0.0%	0.0%	0.0%	0.0%	0.0%	0.0%	0.0%
- with ≥10 CTCs *	1 (5.0%)	100.0%	100.0%	100.0%	100.0%	100.0%	100.0%	100.0%	100.0%	100.0%	100.0%	100.0%
All recurrent/progressive MBC patients	53	98.1%	100.0%	3.8%	100.0%	24.5%	11.3%	20.8%	7.5%	7.5%	37.7%	30.2%
- with CTC count *	51 (96.2%)	98.0%	100.0%	3.9%	100.0%	25.5%	11.8%	21.6%	7.8%	7.8%	39.2%	31.4%
- with 0 CTCs *	19 (37.2%)	100.0%	100.0%	0.0%	100.0%	21.1%	5.3%	10.5%	5.3%	0.0%	26.3%	26.3%
- with 1 CTC *	8 (15.7%)	100.0%	100.0%	0.0%	100.0%	0.0%	0.0%	25.0%	0.0%	0.0%	25.0%	0.0%
- with 2–4 CTCs *	11 (21.6%)	90.9%	100.0%	0.0%	100.0%	36.4%	27.3%	9.1%	9.1%	9.1%	45.5%	45.5%
- with 5–9 CTCs *	3 (5.9%)	100.0%	100.0%	0.0%	100.0%	0.0%	0.0%	33.3%	0.0%	0.0%	33.3%	0.0%
- with ≥10 CTCs *	10 (19.6%)	100.0%	100.0%	20.0%	100.0%	50.0%	20.0%	50.0%	20.0%	30.0%	70.0%	60.0%

Figure S2. Gene expression in samples with and without detected CTCs in corresponding cytology slides.

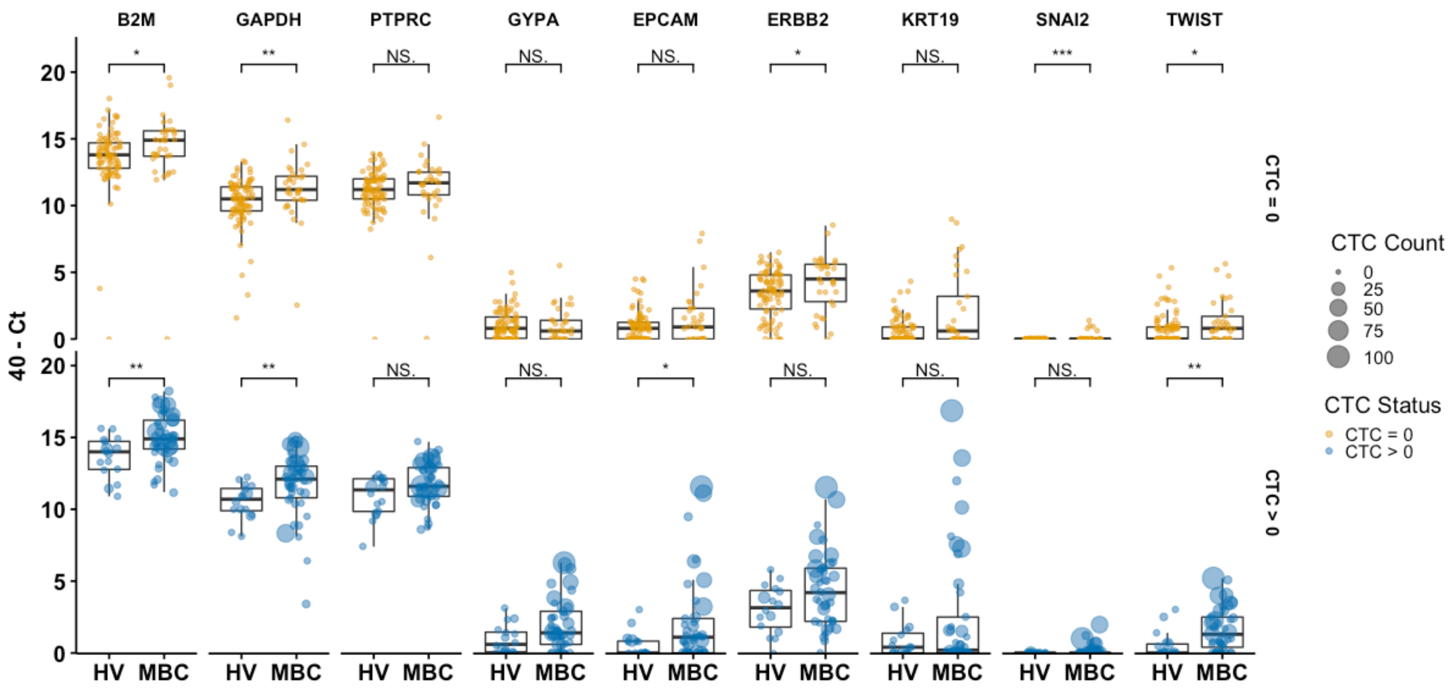

Figure S3. CTC-related gene expression in newly diagnosed and recurrent/progressive metastatic breast cancer (MBC). (**a**) There was no difference in expression between newly diagnosed MBC and recurrent/progressive MBC. (**b**) Differences between MBC cohorts and HV.

a) **b)**

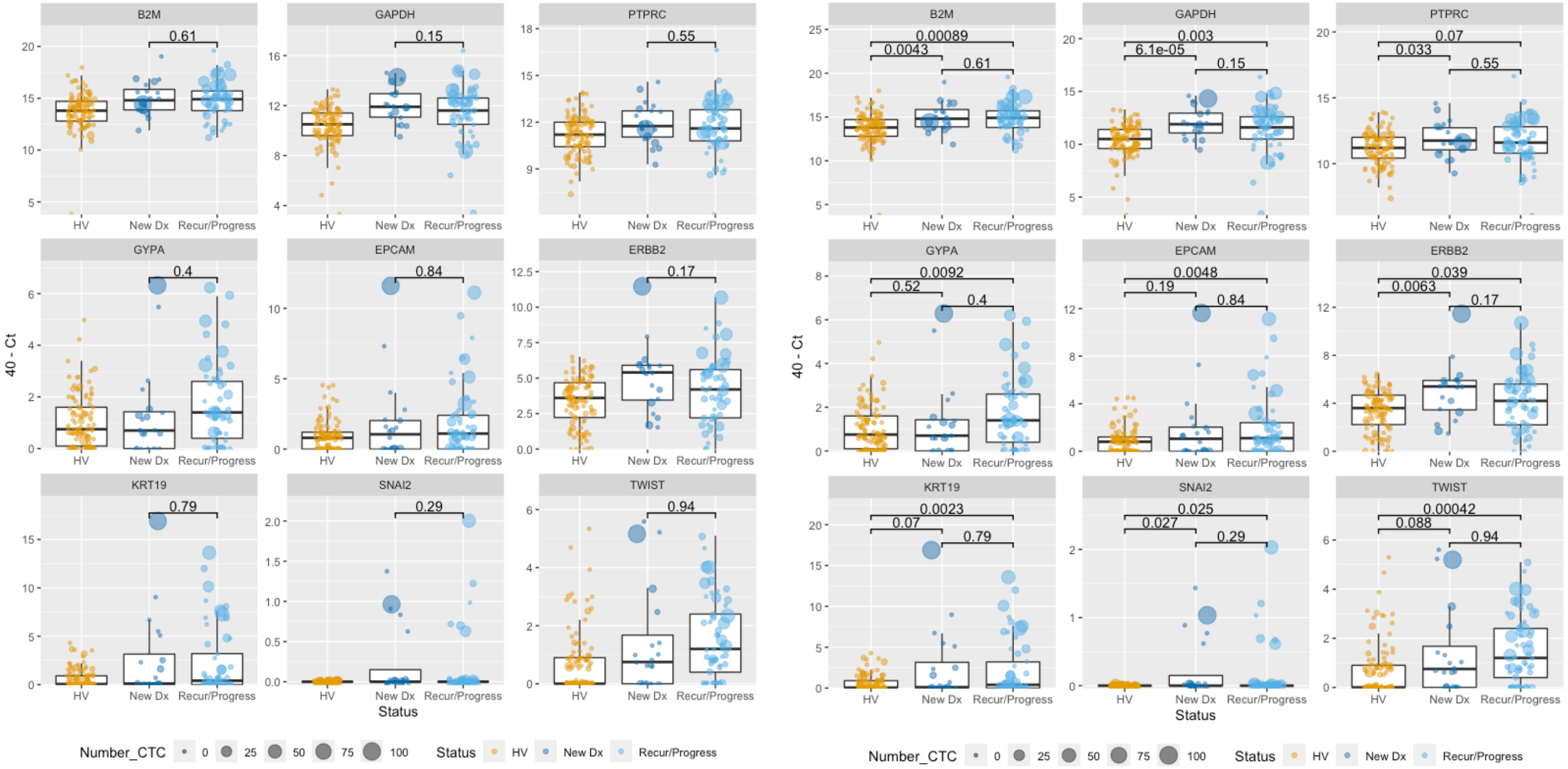

Figure S4. CTC count and CTC-related gene expression hierarchical clustering. Here we can see the highest expression of CTC-related gene expression was in a cluster that includes triple-negative breast cancer (TNBC). The highest expression also includes several samples that had no CTCs detected by cytopathology evaluation. SNAI2 expression is seen in both samples with CTCs and samples with no CTCs detected by cytopathology evaluation.

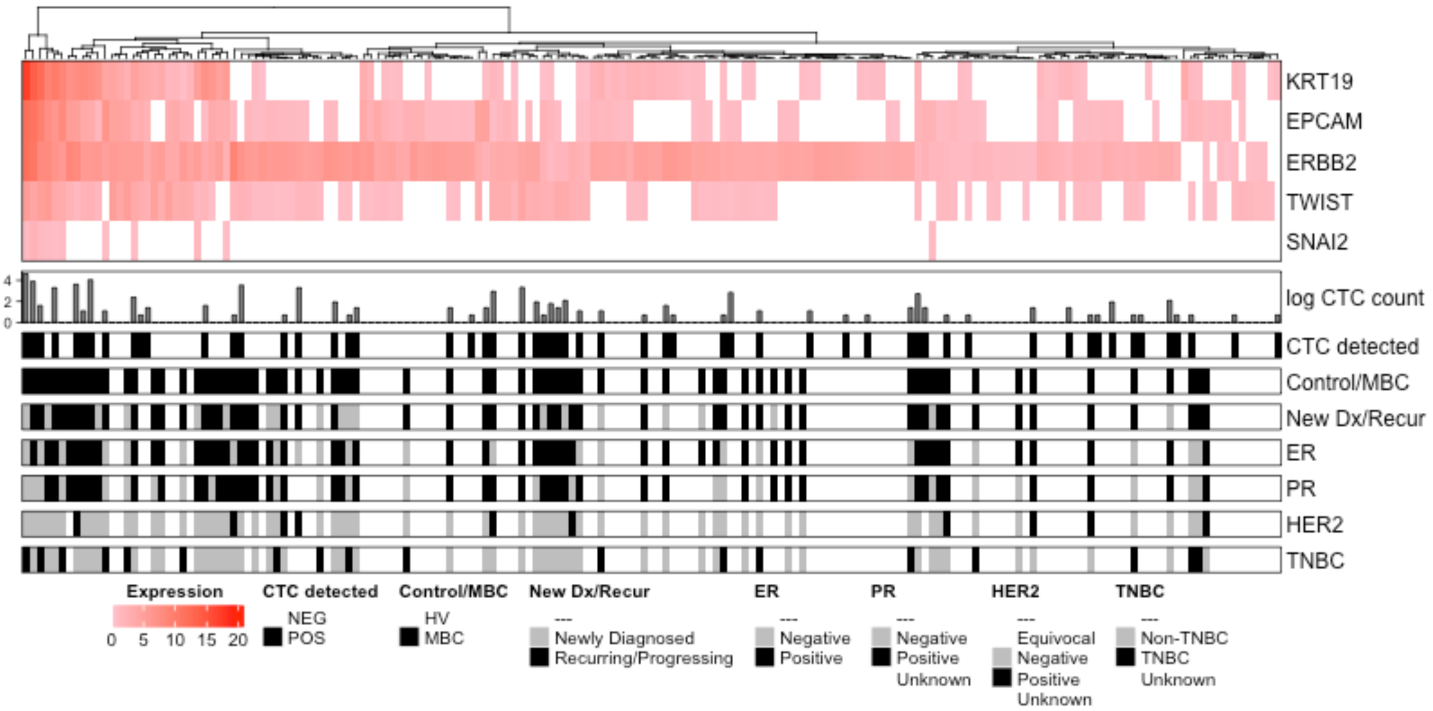

Figure S5. Trend for higher CTC-related gene expression from samples collected from a port, VP, venipuncture.

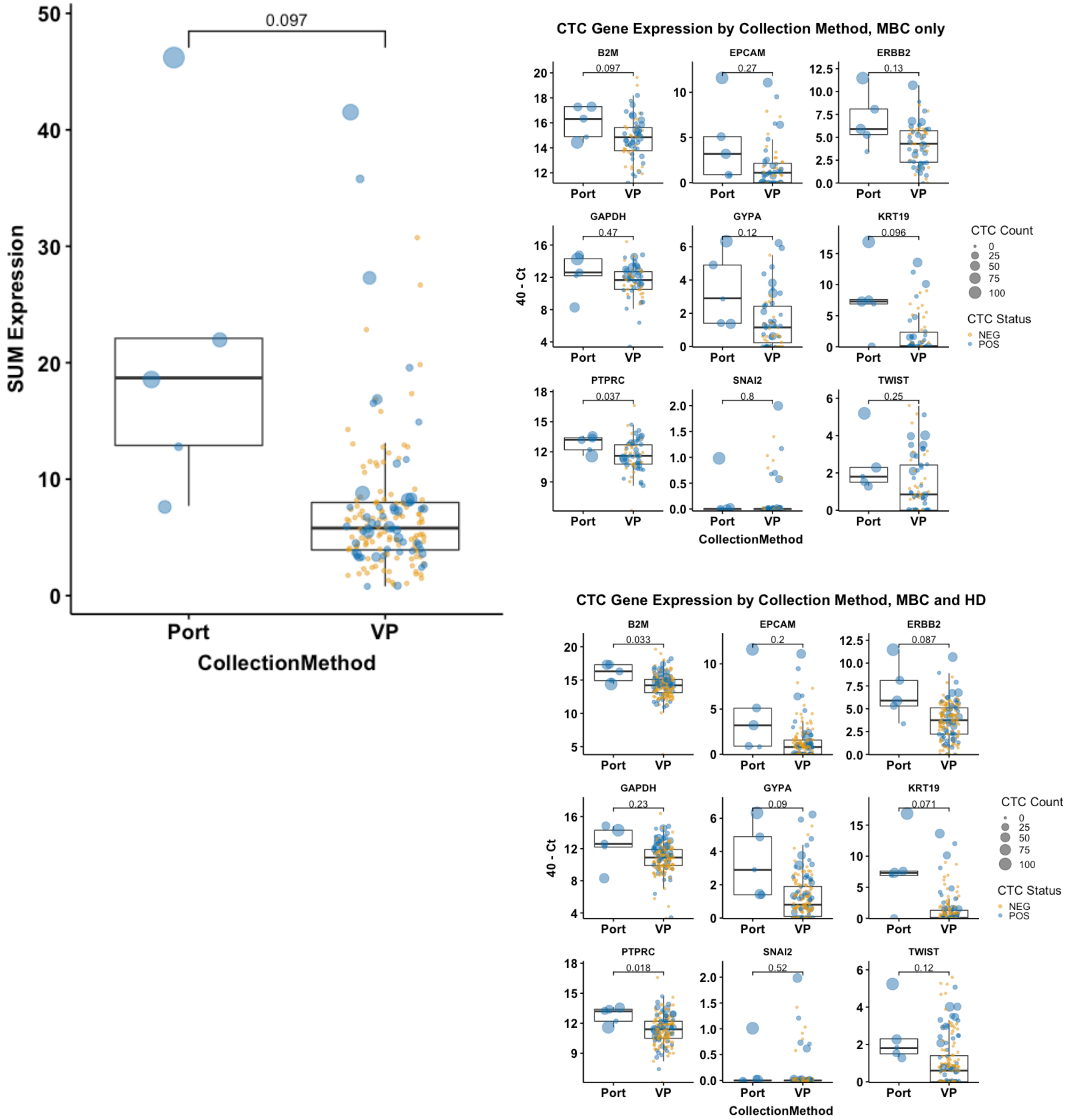

Figure S6. CHAID (Chi-squared automatic interaction detection) decision tree shows that among the samples tested by quantitative polymerase chain reaction, none from HVs had both more than 1 observable CTC by cytopathological review and at least 1 CTC-related gene with high expression (Ct < 35).

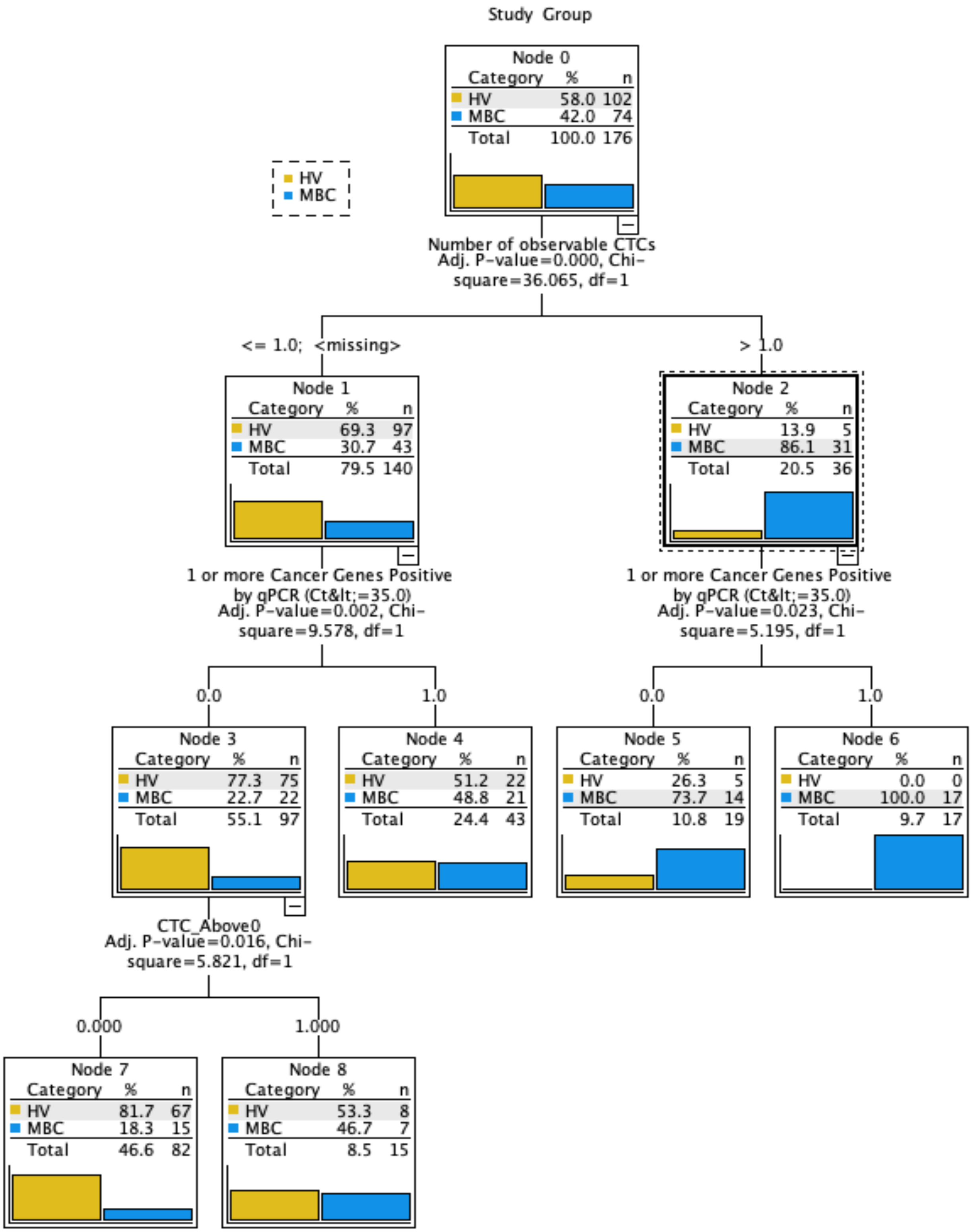

Figure S7. (**a**) Genes from the KEGG Cancer Pathway were differentially expressed between harvests from HER2− (n=8) and HER2+ (n=6) MBC patients ($p < 0.05$). (**b**) Net TPM scores of these 10 genes for HER2−, HER2+, and unknown-status samples (n=2).

(a)

GNG2
DCC
RASGRP2
STK4
F2
COL4A1
IL12B
CAMK2B
EPO
SMO

(b)

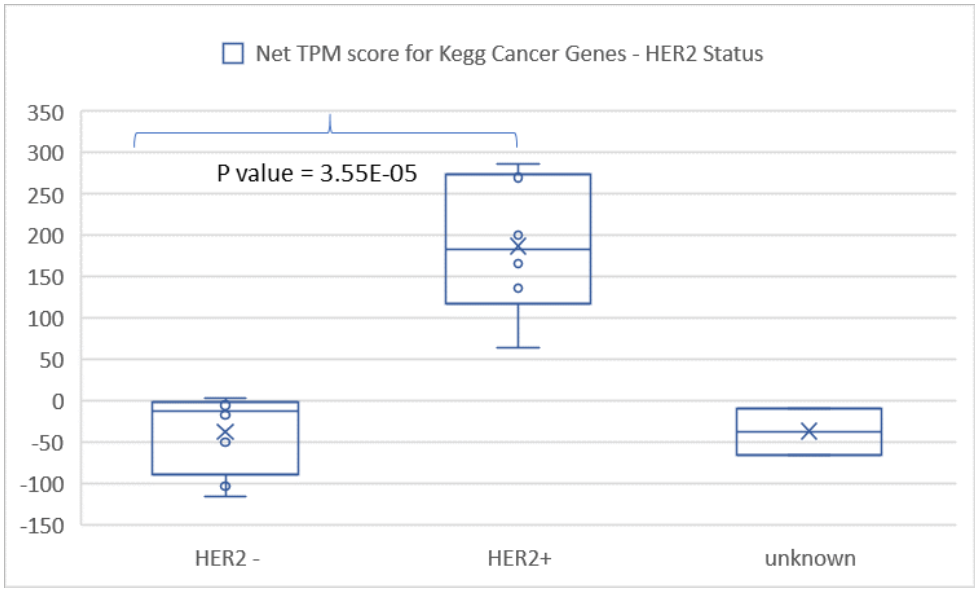

Figure S8. (**a**) Genes from the KEGG Cancer Pathway were differentially expressed ($p < 0.05$) between harvests from MBC patients with the absence (n=6) or presence (n=10) of metastasis to the lymph nodes. (**b**) Net TPM scores of these 18 genes according to the absence or presence of lymph metastases in MBC patient harvests.

(a)

GNG5
NCOA1
TXNRD2
IL6
MSH6
AR
FGFR3
PTGER3
RALB
ELOB
EGLN1
ELK1
GNAI1
TCF7
TRAF1
BMP2
IL7R
ESR1

(b)

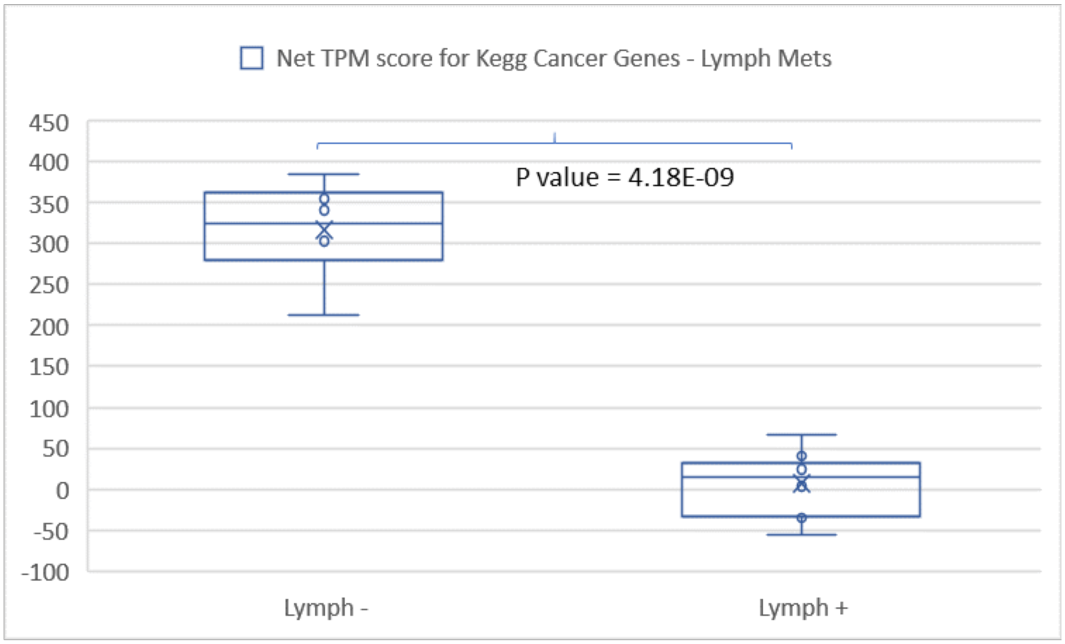

Figure S9. (**a**) Genes from the KEGG Cancer Pathway were differentially expressed ($p < 0.05$) between harvests from MBC patients with the absence (n=6) or presence (n=10) of bone metastasis. (**b**) Net TPM scores of these 16 genes according to the absence or presence of bone metastases in MBC patient harvests.

(a)

ARHGEF1
CASP7
FZD1
CTBP2
BID
CALML6
DAPK1
FN1
PPARD
NOTCH2
JAG2
JUP
TRAF5
CYCS
PRKACB
GNA11

(b)

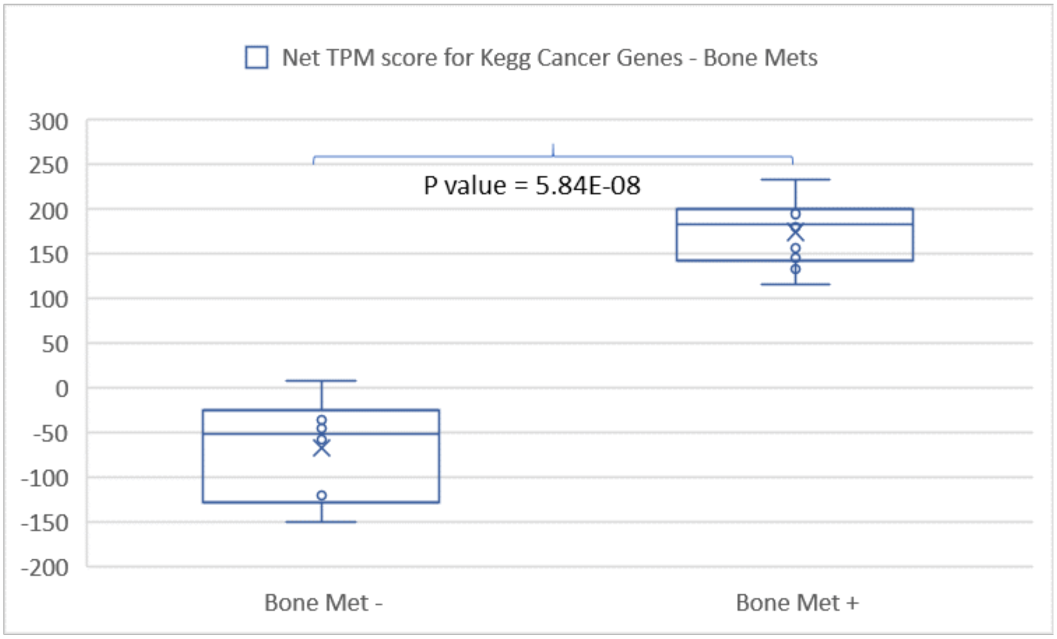

Table S2. Summary of CTC counts on HER2 FISH slides for eligible healthy volunteers (HVs) and metastatic breast cancer (MBC) patients with evaluable HER2 FISH results

ANG-002 HOMING Study No. of CTCs on FISH Slide	Evaluable HVs	All MBCs	Evaluable MBC Patients	
			Newly Diagnosed	Recurring/ Progressing
**0 CTCs**	33 (86.8%)	73 (72.3%)	31 (68.9%)	42 (75.0%)
**1 CTC**	5 (13.2%)	22 (21.8%)	10 (22.2%)	12 (21.4%)
**2-3 CTCs**	0 (0.0%)	6 (5.9%)	4 (8.9%)	2 (3.6%)
**Total**	38	101	45	56
**Fisher exact p-value**	0.149		0.568	
